# Aqueous *Cymbopogon citratus* Extract Mediated Silver Nanoparticles: Part I. Influence of Synthesis Parameters, Characterization, and Biomedical Studies

**DOI:** 10.3390/nano15050328

**Published:** 2025-02-20

**Authors:** Himabindu Kurra, Aditya Velidandi, Mounika Sarvepalli, Ninian Prem Prashanth Pabbathi, Vikram Godishala

**Affiliations:** 1 Department of Biotechnology, Vaagdevi Degree and P.G. College, Warangal 506001, India; bindukurra21983@gmail.com; 2 Department of Biotechnology, Bharatiya Engineering Science and Technology Innovation University, Gorantla 515231, India; 3 Department of Biotechnology, National Institute of Technology, Warangal 506004, India; aditya.velidandi@gmail.com (A.V.); mouni.sarvepalli@gmail.com (M.S.); ninian86@gmail.com (N.P.P.P.)

**Keywords:** biomedical applications, green synthesis, silver nanoparticles, synthesis parameters

## Abstract

This study explores the green synthesis of silver nanoparticles (AgNPs) using *Cymbopogon citratus* (lemongrass) extract as a reducing agent. Synthesis was confirmed by a color change (light yellow to dark brown) under optimal conditions: 1.50 mM silver nitrate, 3.5% *v*/*v* extract, at 100 °C, with a pH of 9, and for 60 min. The AgNPs exhibited spherical morphology, a hydrodynamic diameter of 135.41 ± 49.30 nm, a zeta potential of −29.9 ± 1.4 mV, crystalline structure, and minimal aggregation. AgNPs showed significant antibacterial activity, particularly at >20 µg/well, with the zones of inhibition varying by bacterial strain. In vitro studies demonstrated anti-inflammatory, antidiabetic (α-glucosidase and α-amylase inhibition), and antioxidant activities, with AgNPs outperforming plant extract and nearing standard efficacy at higher concentrations. Cyto-toxicity studies indicated that AgNPs and plant extract were less toxic than doxorubicin but exhibited concentration-dependent effects on cancerous and non-cancerous cells. Eco-toxicity assays revealed that AgNPs were less acutely toxic than controls but posed risks with prolonged exposure. This work highlights the eco-friendly synthesis of AgNPs and their potential in biomedical applications, demonstrating efficacy in antibacterial and antioxidant activities.

## 1. Introduction

Extensive research is conducted on nanoparticles (NPs) owing to their size-related benefits, stability, strength, activity, substantial surface area, and distinctive chemical and biological characteristics [1,2]. These attributes offer a broad range of applications in nanomedicine: their use as antimicrobial agents, in wound dressing, in the treatment of diabetic wounds, for diagnostic purposes, targeted drug delivery, and their role in biomedical applications as biosensors [3,4]. Transitional metal NPs like silver (Ag) [5], gold [6], zinc [7], copper [8], iron [9], palladium [10], and platinum [11] are receiving increased attention because of their versatile characteristics and diverse applications, particularly in serving as catalytic and antimicrobial agents [12,13].

Silver nanoparticles (AgNPs), in particular, have garnered attention in the field of nanotechnology due to their economical nature, non-toxic attributes, environmental friendliness, and expansive surface area [14]. Compared to other noble and transitions metals, Ag is a cost-effective choice [15]. The plasmonic impact of Ag finds widespread application in catalyst-related activities [16]. The synthesis process plays a crucial role in determining the size and shape of AgNPs, which subsequently influences their functional properties [17]. An examination of the literature reveals that the removal percentage and reaction time in the photocatalytic process are contingent upon the size of the AgNPs utilized [18]. Various methods exist for AgNPs’ synthesis, including chemical reduction, mechanical synthesis, and biological approaches [19].

In the context of biological applications, NPs synthesized using *Cymbopogon citratus* extract have shown promising antioxidative, anti-inflammatory, and antidiabetic properties [20,21,22,23,24]. These biological activities are particularly significant, given the growing interest in developing multifunctional AgNPs that can address complex health challenges, such as chronic inflammation, oxidative stress, and metabolic disorders [25,26]. The antioxidative properties of AgNPs are largely attributed to their ability to scavenge free radicals, thereby preventing cellular damage and reducing the risk of chronic diseases such as cancer and cardiovascular disorders [27,28]. AgNPs have shown promising potential in anti-inflammatory applications, with one key mechanism being their ability to inhibit protein denaturation, a process linked to inflammation [29]. Moreover, the antidiabetic potential of AgNPs is linked to their ability to modulate glucose metabolism, possibly through the inhibition of carbohydrate-digesting enzymes [30].

The present work focuses on the green synthesis of AgNPs using aqueous *C. citratus* extract (ACCE) to exploit an economical green synthesis and their potential in biomedical applications. Table 1 presents the details of the literature that reported on AgNPs synthesized using *C. citratus* extract. From the literature, it was evident that the *C. citratus*-synthesized AgNPs were not explored for different biomedical applications such as anti-inflammation. Furthermore, the toxicity of the AgNPs was also underreported (Table 1).

The objectives of this work were as follows: (i) to study the influence of various synthesis parameters using the one-factor-at-a-time (OFAT) approach; (ii) the characterization of AgNPs synthesized using standard analytical techniques (ultraviolet-visible spectroscopy (UV-Vis spec), dynamic light scattering (DLS), zeta potential (ZP), X-ray diffraction (XRD), field emission scanning electron microscope (FE-SEM) coupled with energy-dispersive X-ray spectroscopy (EDX), and a transmission electron microscope (TEM)); (iii) the evaluation of their in vitro biological activities, such as antibacterial, anti-inflammatory, antidiabetic, and antioxidative effects; and (iv) the cyto-toxicity and *Artemia nauplii* eco-toxicity of the AgNPs to provide a comprehensive assessment of their potential as therapeutic agents.

## 2. Materials and Methods

### 2.1. Materials

#### 2.1.1. Chemicals Used

All chemicals used and 0.22 μm polyvinylidene fluoride (PVDF) syringe filters were purchased from HiMedia laboratories Pvt. Ltd. (Hyderabad, India). All chemicals were >99% pure. Distilled water (DW; pH was in the range of 6.6 to 7.4) was used for the work. The pH of solutions was adjusted by using 1 M NaOH and 1 N HCl, accordingly, wherever required. The preparation of stock solutions and necessary dilutions was made using DW. Non-ionic detergent was used to wash the glassware, later rinsed with DW several times, and then dried in a hot air oven before use.

#### 2.1.2. Plant Collection

Aerial parts (leaves) of *C. citratus* were collected from the local neighborhood, Warangal (Telangana, India) during May 2023. The leaves were of a vibrant light green color indicating the early stages of development, healthy growth, and vitality. *C. citratus* leaves were washed several times with DW, cleaned with ethanol, and later shade dried under sterile conditions by keeping them at room temperature (RT). The dried leaves were made into fine powder using a domestic mixer.

### 2.2. Synthesis of AgNPs

#### 2.2.1. Extract Preparation

Extract (ACCE) was prepared by mixing 6 g of fine powder in 100 mL of DW and kept for 2 days in an orbital shaker incubator (REMI, Mumbai, India) at 37 °C. After incubation, the extract was passed through Whatman filter paper no. 1 (Sigma-Aldrich Ltd., St. Louis, MO, USA) followed by 0.22 µm PVDF syringe filters and stored for further use at 4 °C.

#### 2.2.2. Synthesis

**Figure 1:**
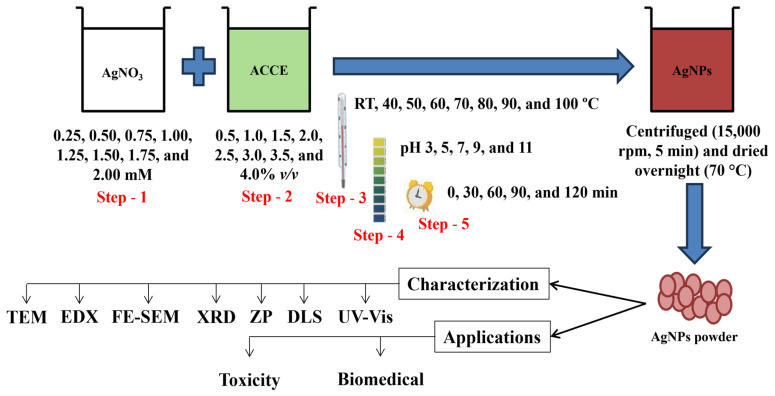
Schematic representation of this study.

AgNO_3_ solution was used as a Ag precursor source for the AgNPs formation. The synthesis reaction was extracted (ACCE; % *v*/*v*) in 1 mL of AgNO_3_ solution. Initial experiments were carried out in 1.5 mL micro-centrifuge tubes at RT for 30 min. A change in color from colorless to pale yellow/dark brown indicated the occurrence of AgNPs, and UV-Vis spec was used from 300 to 700 nm to monitor the AgNPs formation after 30 min of incubation time. Later, centrifugation was conducted for 5 min at 15,000 rpm (RCF: 20,630× *g*; KUBOTA 3300; Table-top Micro Centrifuge; RA-2024; Osaka, Japan) to separate the AgNPs. Using DW, the obtained pellets were cleansed and centrifuged. This step was repeated thrice to remove excess extract and Ag precursor from the reaction solution. Then, pellets were kept at 70 °C overnight in a hot air oven. Dried pellets were scrapped and used for characterization and further work. Figure 1 presents the schematic representation of work.

#### 2.2.3. Influence of Synthesis Parameters

To study the influence of various parameters in the synthesis of AgNPs, five influencing factors were considered: Ag precursor (as a metal ion source; AgNO_3_) concentration, extract (as reducing, capping, and stabilizing agents; ACCE) concentration, reaction temperature, reaction pH, and reaction incubation time for the synthesis. The influence of the AgNO_3_ concentration on the synthesis was studied by varying the concentrations from 0.25, 0.50, 0.75, 1.00, 1.25, 1.50, 1.75, and 2.00 mM. Extract concentration was fixed at 3% *v*/*v*. The reaction temperature and reaction time for the synthesis were RT and 30 min, respectively. To determine the influence of extract concentration (ACCE) on the AgNPs synthesis, varying volumes (concentrations) were used: 0.5, 1.0, 1.5, 2.0, 2.5, 3.0, 3.5, and 4.0% *v*/*v*. The reaction temperature and reaction time for the synthesis were RT and 30 min, respectively. The observed AgNO_3_ concentration from a previous experiment was used. The influence of the reaction temperature on the synthesis of AgNPs was studied by varying the reaction temperature of the synthesis from RT, 40, 50, 60, 70, 80, 90, and 100 °C. The reaction time for the synthesis was 30 min. AgNO_3_ and extract concentrations from previous experiments were used. To study the influence of reaction pH on the synthesis of AgNPs, the reaction pH of the system was adjusted to 3, 5, 7, 9, and 11. The reaction time for the synthesis was 30 min, and the observed reaction temperature and AgNO_3_ and extract concentrations from previous experiments were used. The influence of the reaction time on the synthesis of AgNPs was studied by varying the reaction time from 0, 30, 60, 90, and 120 min. The observed values of reaction temperature, reaction pH, AgNO_3,_ and extract concentrations from previous experiments were used.

### 2.3. Characterization of AgNPs

UV-Vis spec (Model: UV-1800; Manufacturer: Shimadzu, Kyoto, Japan) was used to record the absorbance spectra from 300 to 700 nm for all synthesis experiments. The particle size distribution (PSD) and zeta potential (ZP) analysis of the synthesized AgNPs was studied using a particle analyzer (Model: LiteSeizer 500; Manufacturer: Anton Paar, Graz, Austria) [38]. DLS is a technique used to determine the PSD and polydispersity index (PDI) of AgNPs in solution. It measures the fluctuations in light scattering due to Brownian motion, providing insights into the hydrodynamic diameter of the AgNPs. ZP assesses the surface charge of AgNPs, indicating their stability in suspension. A higher absolute value of ZP signifies stronger electrostatic repulsion between particles, which can prevent aggregation and enhance colloidal stability [38]. XRD (Model: X’Pert Pro; Manufacturer: Malvern PANalytical, Malvern, UK) was used for analyzing the crystalline structure of AgNPs. It provides information about the phase purity, crystallite size, and lattice parameters by examining the diffraction patterns produced when X-rays interact with the crystal lattice of the AgNPs. The approximate crystallite size can be determined using Debye–Scherrer’s equation (Equation (1)), which is recognized as one of the most fundamental and commonly used methods for calculating particle size, utilizing the 2θ and FWHM values obtained from the XRD data [39]. AgNPs powder was placed on a carbon grid, gold coated using a sputter coater. Then, the surface morphology, size, and shape were observed using FE-SEM (Hitachi S-4500; Chiyoda, Japan). Element composition was studied by using EDX coupled with FE-SEM. TEM (Model: Tecnai 10; Manufacturer: Philips, Amsterdam, The Netherlands) allows for the high-resolution imaging of AgNPs, providing detailed information on their morphology, size, and distribution. This technique involves transmitting electrons through a thin sample, enabling the visualization of the internal structure and the shape of AgNPs at the nanoscale [40].

(1) $$D\;\left(nm\right)=\frac{k\lambda }{\beta \cos\theta }$$

where *D* is the size of the crystallites in nm; *κ* is the shape factor of the AgNPs, which is 0.94; λ is the X-ray wavelength of Cu Kα, which is 0.154 nm; *β* is the FWMH measured in radians; and *θ* is the Bragg angle of the peaks measured in radians. The peak position (2*θ*) and FWMH were determined by using OriginPro 2024b software (Northampton, MA, USA).

### 2.4. Biomedical Studies

#### 2.4.1. AgNPs Stock Solution

AgNPs stock solution was prepared by adding 1 mg of AgNPs in 1 mL of DW. Furthermore, the solution was sonicated (20% amplitude, 10 min, pulse on/off for 5 s) for the uniform distribution of AgNPs in the solution. The AgNPs were sonicated under the same conditions before use. All stock solutions were prepared freshly and used.

#### 2.4.2. Antibacterial Studies—Agar Well Diffusion Assay

A total of 9 bacterial (*Acinetobacter baumannii*, *Bacillus subtilis*, *Enterococcus faecalis*, *Escherichia coli*, *Klebsiella pneumoniae*, *Micrococcus luteus*, *Proteus vulgaris*, *Pseudomonas aeruginosa*, and *Staphylococcus aureus*) species were used. The cultures were provided by Dr. Venkataramana Kandi (Assistant Professor), Department of Microbiology, Prathima Institute of Medical Sciences, Karimnagar (Telangana, India). The cultures obtained were regularly sub-cultured in nutrient agar plates and upon requirement, pre-inoculum was prepared in nutrient broth.

In this in vitro method, agar plates inoculated with bacterial strains were prepared, and wells were made using sterile well borer (diameter 6 mm). AgNPs (stock solution of 1 mg/mL) were loaded at different volumes (10 µL, 20 µL, 30 µL, 40 µL, and 50 µL) and labeled (10 µg, 20 µg, 30 µg, 40 µg, and 50 µg) accordingly. Standard antibiotics (Amp, Chl, Km, and Pen) were loaded at 10 µL (stock solution of 1 mg/mL). The plates were then incubated, typically at 37 °C for 24 h, allowing the AgNPs to diffuse into the agar. The antibacterial activity is indicated by a clear zone of inhibition (ZOI) surrounding the wells, where bacterial growth has been prevented. The size of these ZOIs correlates with the effectiveness of the AgNPs against the test bacteria [40].

#### 2.4.3. Anti-Inflammatory Studies

The assay evaluates the ability of the synthesized AgNPs to inhibit protein denaturation, which is an important anti-inflammatory mechanism. Protein denaturation often correlates with inflammation, so compounds that prevent or inhibit this process are considered to have anti-inflammatory properties. Fresh chicken egg white is mixed with 0.5 M Tris-HCl buffer (pH 7.07) at a 1:4 ratio to reduce its viscosity. Each reaction tube contains: 0.2 mL of egg white solution, 1.8 mL of Tris-HCl buffer, and various concentrations of AgNPs. Incubate the reaction mixture for 15 min at 37 °C. Followed by heating the mixture at 80 °C for 5 min in a water bath. Cool the samples and measure their absorbance at 660 nm. Ibuprofen (Ibu) and ACCE were used as positive and negative controls, respectively. The protein denaturation inhibition (PrDI) percentage was calculated using Equation (2) [41].

(2) $$\mathrm{PrDI}\;\%=\left(\frac{I_{c}-I_{t}}{I_{c}}\right)\;\times \;100$$

where *I_c_* and *I_t_* were the absorbance of blank (without sample) and sample, respectively.

#### 2.4.4. Antidiabetic Studies

##### α-Glucosidase Assay

p-nitrophenyl-α-D-glucopyranoside (pNGP) was used to evaluate α-glucosidase enzyme activity, which plays a role in carbohydrate digestion. To study the inhibition of α-glucosidase by AgNPs, the reaction mixture was prepared as follows: 10 µL of α-glucosidase enzyme (a 1 mg/mL stock solution) was mixed with 20 µL of various concentrations of AgNPs (10, 20, 30, 40, and 50 µg/mL). The mixture was incubated at RT for 15 min to allow interaction. After incubation, 40 µL of the pNGP substrate (a 1 mg/mL stock solution) was added to the mixture, which was then incubated at 37 °C for 5 min to allow the enzyme to act on the substrate. The reaction volume was adjusted to 200 µL by adding Tris-HCl buffer, and the absorbance was immediately measured to assess the residual enzyme activity, which reflects the enzyme’s glycolytic potential and the inhibitory effect of AgNPs. The experiment was performed using a 96-well flat-bottom microplate to allow multiple tests simultaneously. Absorbance values were recorded at 415 nm using a BIORAD Model 680 microplate reader (Hercules, CA, USA), which also had temperature control. Acarbose (Acr) and ACCE were used as positive and negative controls, respectively. The α-glucosidase inhibition percentage was calculated using Equation (3) [41].

(3) $$\mathrm{Inhibition}\;\%=\left(\frac{D_{c}-D_{t}}{D_{c}}\right)\times 100$$

where *D_c_* and *D_t_* were the absorbance of blank (without sample) and sample, respectively.

##### α-Amylase Assay

The α-amylase enzyme inhibitory activity of AgNPs using the dinitro salicylic acid (DNS) method was performed. A 0.5 mL starch solution (0.5% *w*/*v*) was used as the substrate for α-amylase. In total. 0.1 mL of α-amylase (a 1 mg/mL stock solution) was added to the starch solution. The mixture was incubated at 37 °C for 10 min to allow the enzyme to break down the starch. After incubation, 0.2 mL of DNS reagent was added to stop the reaction and to develop a color indicating the extent of starch breakdown. For assessing the inhibitory activity of AgNPs, initially, the α-amylase enzyme was incubated with 0.1 mL of various concentrations of AgNPs (10, 20, 30, 40, and 50 µg/mL) for 30 min. The reaction tubes were placed in a boiling water bath for 5 min, after which they were cooled to RT. To dilute the color product, 3 mL of DW was added to the reaction tubes. The absorbance of the solution was measured at 540 nm to quantify the α-amylase activity based on the color intensity of the reaction product. Acr and ACCE were used as positive and negative controls, respectively. The α-amylase inhibition percentage was calculated using Equation (3) [40].

#### 2.4.5. Antioxidative Studies

##### DPPH Assay

A typical reaction tube contained 1.9 mL of 2,2-diphenyl-1-picrylhydrazyl (DPPH) solution (1.5 × 10^−4^ M in methanol); a stable free radical used to evaluate antioxidant activity. AgNPs were added to the reaction tubes in incremental volumes (20, 40, 60, 80, and 100 μL) from a stock solution (1 mg/mL). This resulted in final concentrations of 20, 40, 60, 80, and 100 μg/mL in the reaction tube. The total reaction volume was brought to 2 mL by adding DW. The reaction contents were stirred and incubated in the dark at 37 °C for 30 min to ensure the interaction between the AgNPs and DPPH. After incubation, the absorbance of the reaction tubes was measured at 517 nm to assess the remaining DPPH radicals. Ascorbic acid (AscA) and ACCE were used as positive and negative controls, respectively. The DPPH inhibition percentage was calculated using Equation (4) [41].

(4) $$\mathrm{Inhibition}\;\%=\left(\frac{O_{c}-O_{t}}{O_{c}}\right)\times \;100$$

where *O_c_* and *O_t_* were the absorbance of blank (without sample) and sample, respectively.

##### ABTS Assay

Initially, 300 mg of 2,2′-azino-bis(3-ethylbenzothiazoline-6-sulfonic acid (ABTS) was dissolved in 100 mL of DW to prepare the stock solution. ABTS^•+^ free radicals were created by mixing the ABTS solution with potassium persulfate (2.5 mM) in equal volumes. This mixture was incubated in the dark for 24 h to generate stable ABTS^•+^ radicals. In total, 20 mL of the ABTS^•+^ solution was diluted with DW until the absorbance reached approximately 0.8 at 734 nm. For each reaction, 2 mL of the diluted ABTS^•+^ solution was mixed with 0.1 mL of various concentrations of AgNPs (20, 40, 60, 80, and 100 μg/mL). The reaction mixtures were incubated in the dark for 10 min to allow interaction between the AgNPs and ABTS^•+^ radicals. After incubation, the decrease in color intensity (due to the reduction in ABTS^•+^ radicals) was measured at 734 nm. AscA and ACCE were used as positive and negative controls, respectively. The ABTS^•+^ inhibition percentage was calculated using Equation (4) [40].

##### TRP Assay

The reaction mixture of the total reducing power (TRP) assay consisted of 0.1 mL of various concentrations of AgNPs (20, 40, 60, 80, and 100 μg/mL), 2 mL of sodium phosphate buffer (100 mM, pH of 6.5), and 2 mL of 1% potassium ferricyanide. This mixture was incubated for 20 min at 50 °C, allowing ferricyanide to be reduced to ferrocyanide. After incubation, 2 mL of 10% trichloroacetic acid was added to stop the reaction. The mixture was then centrifuged at 1000 rpm for 10 min. After centrifugation, 2 mL of the upper layer (supernatant) was carefully removed and placed in a separate tube. To the 2 mL supernatant, 2 mL of DW and 0.5 mL of ferric chloride were added. The development of a blue color indicated the formation of ferric ferrocyanide, which signifies the reducing power of the AgNPs. The absorbance of the solution was recorded at 700 nm, where a higher absorbance indicates a greater reducing power of the AgNPs. AscA and ACCE were used as positive and negative controls, respectively. The TRP percentage was calculated using Equation (5) [40].

(5) $$\mathrm{Reducing}\;\%=\left(\frac{R_{t}-R_{c}}{R_{t}}\right)\times \;100$$

where *R_c_* and *R_t_* were the absorbance of blank (without sample) and sample, respectively.

### 2.5. Toxicity Studies

#### 2.5.1. Cyto-Toxicity Assay

The cyto-toxicity of the synthesized AgNPs was assessed using cancerous (such as K562, HeLa, A549, HepG2, Caco-2, and B16F10) and non-cancerous (such as MCF-10A and L929) cell lines through a standard MTT assay, following previously published protocol [42]. The cell lines were provided by Dr. Ashok Reddy, Synteny Lifesciences Pvt. Ltd., Hyderabad (Telangana, India). MTT, a yellow cationic tetrazolium salt, penetrates the cytoplasm of metabolically active or viable cells, where it is reduced by mitochondrial dehydrogenase enzymes, leading to the formation of purple formazan precipitate or crystals [41]. The reduction in the tetrazolium salt serves as a toxic indicator for the cells and is used to determine cell viability [43]. The cancer cells were regularly sub-cultured and maintained in RPMI 1640 (Lonza, Basel, Switzerland) media, supplemented with 10% heat-inactivated fetal bovine serum (Gibco, Invitrogen Life Technologies, Waltham, MA, USA) and antibiotics (1% PenStrep; Sigma-Aldrich Ltd., St. Louis, MO, USA). The cells were cultured under conditions of 37 °C in a 5% CO_2_ static incubator (Thermo Fisher Scientific, Waltham, MA, USA). The morphology, growth, and contamination of the cell cultures were routinely monitored using an inverted microscope (Leica, Wetzlar, Germany), and the MTT assay was conducted in 96-well plates.

A cell suspension containing approximately 20,000 cells in 100 µL was prepared and transferred into the wells of a 96-well plate. The plates were incubated for 24 h under controlled conditions (37 °C in a 5% CO_2_ incubator) to allow the cells to adhere and grow. After incubation, 100 µL of freshly prepared AgNPs solutions at increasing concentrations (25, 50, 75, 100, 150, and 200 µg/mL in RPMI 1640 media (HiMedia, Hyderabad, India)) was added to the respective wells, and each well was labeled accordingly. The plates were then incubated for an additional 48 h under the same conditions. Following the second incubation, 20 µL of MTT solution (5 mg/mL in phosphate-buffer saline) was added to each well and incubated for 4 h under the same conditions. This step leads to the formation of purple formazan crystals as a result of viable cell metabolism. After the incubation, the cell suspensions were carefully discarded without disturbing the formed formazan crystals. To dissolve the formazan crystals, 200 µL of dimethyl sulfoxide solvent was added to each well. The absorbance of the wells was then measured at 570 nm using a 96-well plate reader to quantify the amount of formazan produced. Doxorubicin (Dox) and ACCE were used as positive and negative controls, respectively. The cell mortality percentage was calculated using Equation (6) [44].

(6) $$\mathrm{Mortality}\;\%=\left(\frac{M_{c}-M_{t}}{M_{c}}\right)\;\times \;100$$

where *M_c_* and *M_t_* were the absorbance of blank (without sample) and sample, respectively.

#### 2.5.2. Eco-Toxicity Assay

Brine shrimp eggs were incubated in a beaker containing 3% rock salt water for 48 h. Aeration was provided continuously using an air pump. Once the eggs hatched, live *Artemia nauplii* were collected using a Pasteur pipette for the bio-assay. Each bio-assay test tube contained 3 mL of rock salt water and precisely 20 live brine shrimp. Incremental volumes of AgNPs (20, 40, 60, 80, and 100 µg/mL) were added from a stock solution (1 mg/mL) to each test tube to test the AgNPs’ effect at varying concentrations. The bio-assay was conducted at RT with the brine shrimp exposed to the AgNPs. After 24, 36, 48, and 60 h of incubation, the test tubes were observed, and the number of live brine shrimp was recorded. The mortality percentage was calculated using Equation (7) [40].

(7) $$\mathrm{Mortality}\;\%=\left(\frac{\mathrm{Number}\;\mathrm{of}\;\mathrm{dead}}{\mathrm{Initial}\;\mathrm{number}\;\mathrm{of}\;\mathrm{live}}\right)\times \;100$$

### 2.6. Statistical Analysis

All the experiments were performed in triplicates (*n* = 3). Data were presented as mean (*n* = 3) ± standard deviation (error bars). Statistical significance (*p* value < 0.05) was determined using Student’s *t*-test in Microsoft Office 2021. Microsoft Office Excel 2021 and Origin 2024b were used for the graphical representation of results.

## 3. Results and Discussion

### 3.1. Synthesis of AgNPs

The green synthesis of the AgNPs using plant extracts as reducing agents has been the primary focus of several research groups and scientists [3,45,46]. The present work explores the possible application of ACCE as an economical, sustainable, and environmentally friendly reducing agent in the synthesis of AgNPs via biological approach. A change in the color of the reaction solution is the primary indicator in determining the successful synthesis of AgNPs [47,48]. The AgNO_3_ solution was colorless, and the ACCE solution was a light green color. Upon mixing both solutions, the reaction mixture should turn to a light yellow to dark brown color, indicating the presence of AgNPs [47,48]. In the present work, at pre-determined experimental parameters, a light yellow to dark brown (Supplementary Materials) color was observed, which aligns with the literature.

### 3.2. Influence of Synthesis Parameters

The green synthesis of AgNPs is significantly influenced by various parameters such as the concentration of Ag precursor, the type and concentration of the reducing agent, the reaction pH, reaction temperature, and reaction time [22,49,50,51,52,53,54,55]. The concentration of the metal (Ag) precursor determines the size and yield of the AgNPs; higher concentrations typically lead to larger AgNPs [50,52,55]. The reducing agent (extract), often derived from plants, affects the rate of reduction and, consequently, the size and morphology of the AgNPs [40]. Reaction temperature accelerates the reduction process, often leading to smaller AgNPs with a more uniform size distribution [49,51]. Reaction pH plays a crucial role in stabilizing the AgNPs, as it influences the ionization state of the biomolecules in the plant extract, affecting their interaction with Ag^+^ ions [22,53,54]. Finally, reaction time determines the extent of AgNPs growth and agglomeration, with longer times generally leading to larger AgNPs [52,53,54]. The careful optimization of these parameters is essential to control the physicochemical properties of AgNPs.

The height of the peaks (absorbance values) indicates the concentration of AgNPs, while their width reveals information about size distribution. A narrower peak suggests a smaller size range of AgNPs in the solution. Furthermore, as the peak shifts to shorter wavelengths (blue shift), it indicates a decrease in AgNPs size [56,57]. Table 2 presents the OFAT results of the present study. Table 3 presents the comparison with the literature reported.

#### 3.2.1. Influence of Metal Precursor Concentration

The concentration of the metal precursor (AgNO_3_) significantly influences the synthesis of AgNPs, affecting their size, shape, and distribution. Higher precursor concentrations typically lead to an increase in the number of Ag^+^ ions available for reduction, which can result in larger AgNPs due to the increased rate of nucleation and growth [40]. However, excessive precursor concentrations can also cause agglomeration, leading to broader size distributions and less uniform shapes [57]. Conversely, lower precursor concentrations often yield smaller and more uniform AgNPs but may reduce the overall yield [66]. Optimizing the precursor concentration is crucial to achieving desired AgNPs characteristics and enhancing their stability and functionality.

**Figure 2:**
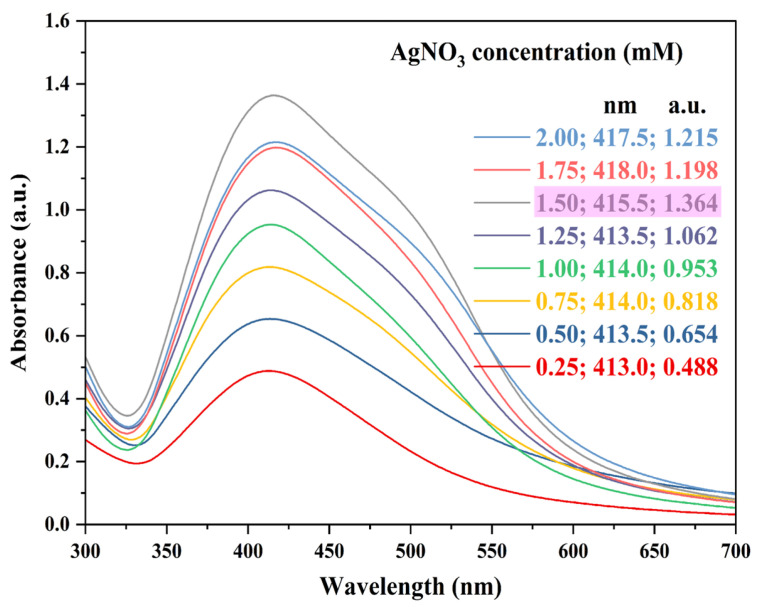
UV-Vis absorption spectra representing AgNPs synthesis at various metal precursor (AgNO_3_) concentrations. Reaction conditions: AgNO_3_ concentration—0.25, 0.50, 0.75, 1.00, 1.25, 1.50, 1.75, and 2.00 mM; ACCE concentration—3.0% *v*/*v*; reaction temperature—RT; reaction pH—6.5 to 7.0; and reaction time—30 min. Pink highlight indicates the Ag precursor concentration chosen for further experiments.

Ag precursor (AgNO_3_) concentration used in the present study was in the range of 0.25 to 2.00 mM. From the results (Figure 2), it was evident that as the concentration of metal (AgNO_3_) precursor increased, the wavelength (i.e., maximum absorption peak) of the samples also increased, causing a red shift [57]. The peak intensity (absorbance values) for the 1.75 mM and 2.00 mM Ag precursor samples was reduced because the AgNPs preferred growth over additional nucleation [60,62]. Therefore, a Ag precursor concentration of 1.50 mM was chosen for further experiments.

#### 3.2.2. Influence of Extract Concentration

The volume of extract used in the process greatly affects AgNPs formation. Plant extracts play a vital role in reducing ions, and using an optimal volume enhances the efficiency of AgNPs formation [58]. The concentration of extract, which contains different phytochemicals at various concentrations, has a significant impact on the average size of AgNPs. This concentration is key for Ag^+^ ions reduction and for producing stable AgNPs at the nanoscale. Hence, optimizing the extract concentration is essential for effective AgNPs synthesis [40,58].

**Figure 3:**
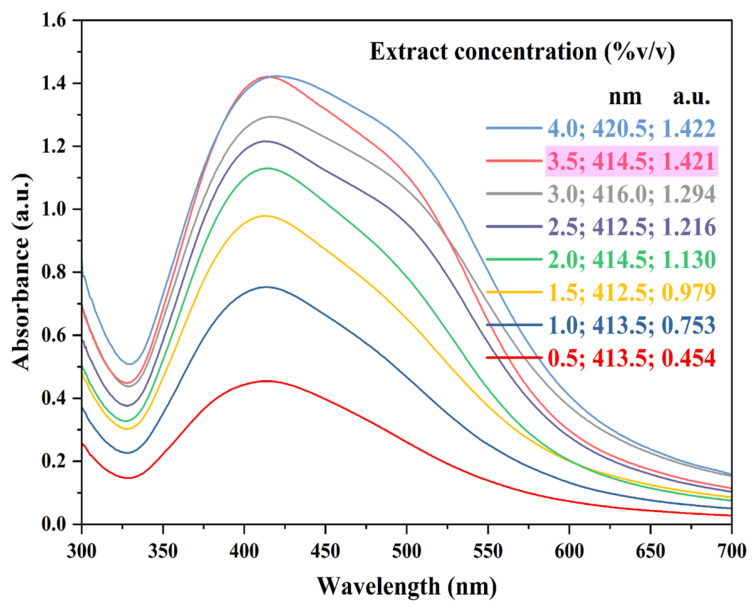
UV-Vis absorption spectra representing AgNPs synthesis at various extract (ACCE) concentrations. Reaction conditions: AgNO_3_ concentration—1.50 mM; ACCE concentration—0.5, 1.0, 1.5, 2.0, 2.5, 3.0, 3.5, and 4.0% *v*/*v*; reaction temperature—RT; reaction pH—6.5 to 7.0; and reaction time—30 min. Pink highlight indicates the extract concentration chosen for further experiments.

Extract (ACCE) concentration used in the present study was in the range of 0.5 to 4.0% *v*/*v*. From the results (Figure 3), it was evident that as the ACCE concentration increased, the wavelength (i.e., maximum absorption peak) of the samples also increased, causing a red shift. An ACCE concentration of 3.5% *v*/*v* was chosen for further experiments.

#### 3.2.3. Influence of Reaction Temperature

Reaction temperature is a critical factor in the synthesis of AgNPs. While the reaction usually occurs at RT, this results in a slower process. However, raising the temperature of the reaction mixture can accelerate the reaction [59]. The typical reaction temperature range for the reaction is between RT and 100 °C. As the reaction temperature increases, the overall conversion rate decreases [67,68], promoting the homogeneous nucleation of Ag nuclei. This process facilitates the production of smaller AgNPs [69,70].

**Figure 4:**
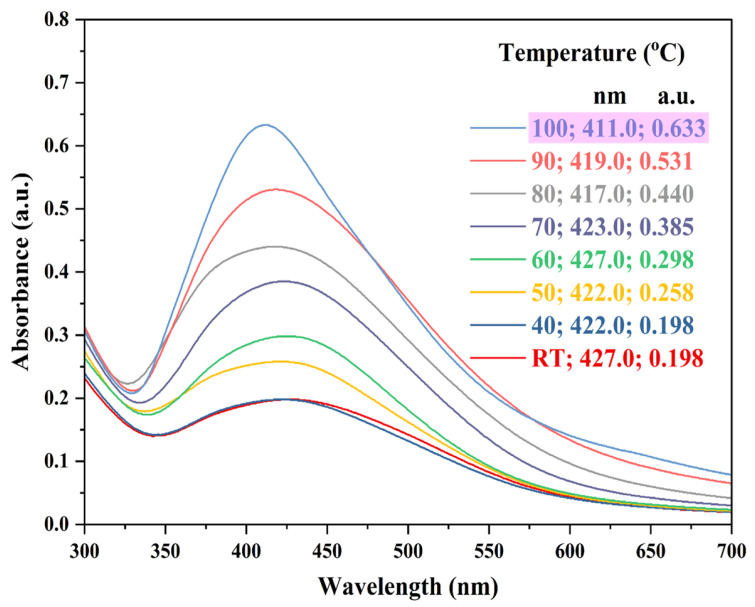
UV-Vis absorption spectra representing AgNPs synthesis at various reaction temperatures. Reaction conditions: AgNO_3_ concentration—1.50 mM; ACCE concentration—3.5% *v*/*v*; reaction temperature—RT, 40, 50, 60, 70, 80, 90, and 100 °C; reaction pH—6.5 to 7.0; and reaction time—30 min. Pink highlight indicates the reaction temperature chosen for further experiments.

The reaction temperature used in the present study was in the range of RT to 100 °C. From the results (Figure 4), it was evident that as the reaction temperature increased, the wavelength (i.e., maximum absorption peak) of the samples decreased, causing a blue shift [59,69,70]. Absorbance values were increased from 0.198 to 0.633 a.u. for RT to 100 °C. Therefore, a reaction temperature of 100 °C was chosen for further experiments.

#### 3.2.4. Influence of Reaction pH

The reaction pH level plays a crucial role in influencing biomolecules, altering their electrical charges, which can hinder their ability to cap and stabilize, ultimately affecting AgNPs growth [71]. In AgNPs synthesis, reaction pH levels determine the properties and reaction kinetics. Different plant extracts contain various bioactive components, e.g., polyphenols, flavonoids, and proteins, which can act as reducing agents but exhibit varying reactivity depending on the reaction pH. Optimizing the reaction pH conditions during synthesis is essential to maximize reduction efficiency and control the size and shape of the AgNPs. Additionally, reaction pH influences the stability and aggregation behavior of the synthesized AgNPs. The surface charge of AgNPs is highly dependent on reaction pH, affecting their colloidal stability and aggregation tendencies [72,73]. By enhancing electrostatic repulsion between AgNPs at certain reaction pH levels, it is possible to prevent or minimize aggregation, which is crucial for achieving uniform and stable dispersions of AgNPs [70,74].

**Figure 5:**
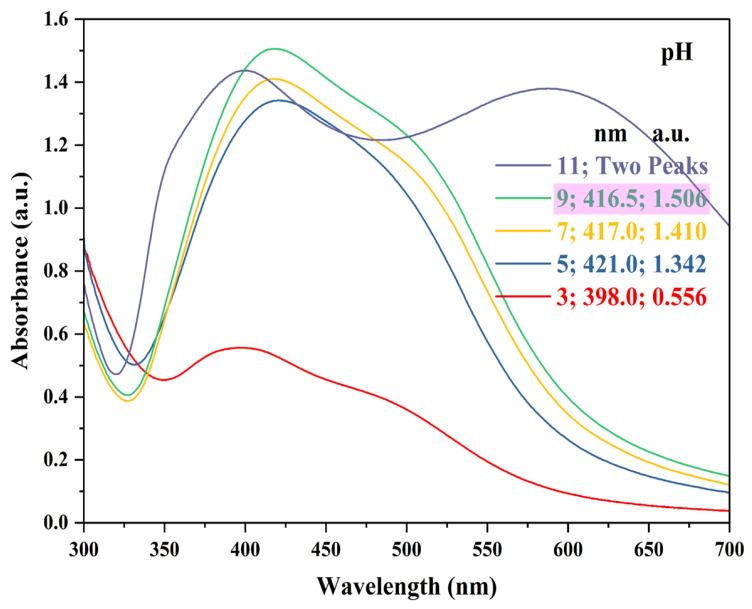
UV-Vis absorption spectra representing AgNPs synthesis at various reaction pHs. Reaction conditions: AgNO_3_ concentration—1.50 mM; ACCE concentration—3.5% *v*/*v*; reaction temperature—100 °C; reaction pH—3, 5, 7, 9, and 11; and reaction time—30 min. Pink highlight indicates the reaction pH chosen for further experiments.

The reaction pH used in the present study was in the range of 3 to 11. From the results (Figure 5), it was evident that as the reaction pH increased, the wavelength (i.e., maximum absorption peak) of the samples decreased, causing a blue shift, overall. At a pH of 3, no visible AgNPs formation, i.e., color change, was observed [56]. Previous studies suggest that anionic repulsion in the solution might be responsible for the slow synthesis and accumulation of AgNPs at lower pH levels [59]. A pH of 11 showed two peaks at 400 nm (1.437 a.u.) and 588 nm (1.38 a.u.). A peak at 400 nm was typically associated with the surface plasmon resonance (SPR) of AgNPs [60,61,62,75]. The SPR is a collective oscillation of free electrons in response to incident light, which enhances the absorption of light at specific wavelengths. A peak at 588 nm may indicate the presence of larger AgNPs or agglomerates. As the size of the AgNPs increases, the SPR peak shifts to longer wavelengths (red shift) [70,76]. The peak at 588 nm can signify the formation of larger clusters or a more complex particle size distribution. The higher pH can also influence the surface charge of the AgNPs, leading to varying degrees of aggregation, which can contribute to the presence of multiple peaks in the spectrum [77]. A similar observation was reported by Handayani et al. [77], when two peaks were observed at a pH of 11 near 422 nm and 673 nm. Therefore, a reaction pH of 9 was chosen for further experiments.

#### 3.2.5. Influence of Reaction Time

The reaction time is essential for AgNPs synthesis, as it ensures an effective interaction between the metal precursor and the reducing agents in the extract [78]. The reaction time starts when the reactant is added to the beaker and continues until the reaction completes or there is a pre-determined reaction time [58]. Plants with higher concentrations of secondary metabolites or phytochemicals are more efficient at reducing the salt (metal precursor). In contrast, plants with fewer reducing compounds take longer to achieve this reduction. However, even plants with lower levels of secondary metabolites can still produce AgNPs quickly. Factors such as the acidity or basicity of the reaction mixture, the reducing strength of the plant extract, light intensity, enzymes, and the presence of secondary metabolites in the extract all influence the reaction time [58].

**Figure 6:**
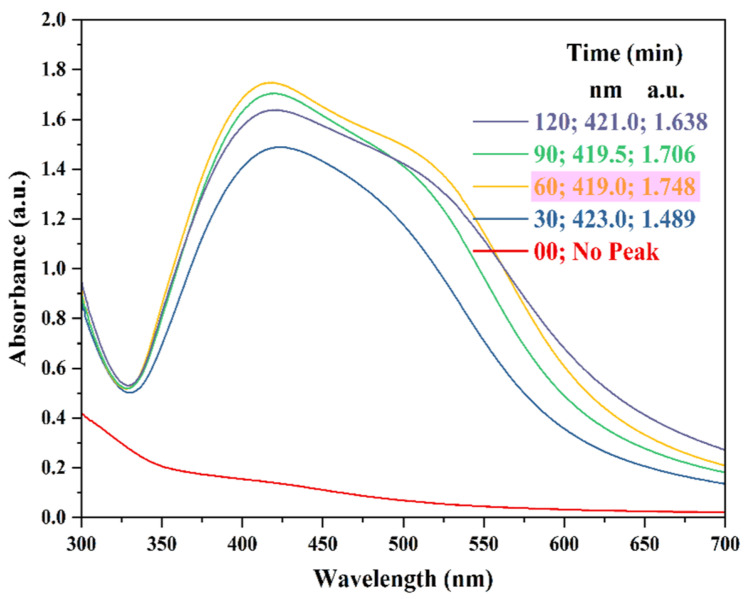
UV-Vis absorption spectra representing AgNPs synthesis at various reaction times. Reaction conditions: AgNO_3_ concentration—1.50 mM; ACCE concentration—3.5% *v*/*v*; reaction temperature—100 °C; reaction pH—9; and reaction time—0, 30, 60, 90, and 120 min. Pink highlight indicates the reaction time chosen for further experiments.

The reaction time used in the present study was in the range of 0 to 120 min. From the results (Figure 6), it was evident that no immediate AgNPs synthesis was observed in the reaction mixture at 0 min. The reduced absorbance peaks observed at longer reaction times (90 and 120 min) were attributed to the aggregation of AgNPs rather than continued nucleation as the reaction time increased [60,62]. Therefore, a reaction time of 60 min was chosen.

AgNPs synthesized at optimal conditions (which were determined in Section 3.2): AgNO_3_ concentration—1.50 mM, ACCE concentration—3.5% *v*/*v*, reaction temperature—100 °C, reaction pH—9, and reaction time—60 min were used for characterization and biomedical studies.

### 3.3. Characterization of AgNPs

#### 3.3.1. Optical Analysis

The emergence of a strong peak in the range of 400 to 440 nm in the absorption spectra confirmed the presence of AgNPs in the reaction solution [60,61,62,75]. This phenomenon, known as Surface Plasmon Resonance, occurs due to the interaction of electromagnetic radiation with the AgNPs’ surface, resulting in a color change [56]. The present study observes the maximum absorption peak in the range of 411 to 427 nm (Table 2), which was in alignment with the literature reported [60,61,62].

#### 3.3.2. Particle Size Distribution

DLS is a widely used technique for determining the PSD and assessing the PDI of NPs and macromolecules in solution. By measuring the fluctuations in scattered light from particles undergoing Brownian motion, DLS provides a comprehensive profile of particle sizes, allowing researchers to identify variations in size within a sample. The PDI, derived from the PSD, quantifies the uniformity of particle sizes, with lower values indicating a more monodisperse population and higher values reflecting greater polydispersity. This non-invasive method is essential, where understanding the size and distribution of colloidal systems is crucial for optimizing their performance and functionality.

**Figure 7:**
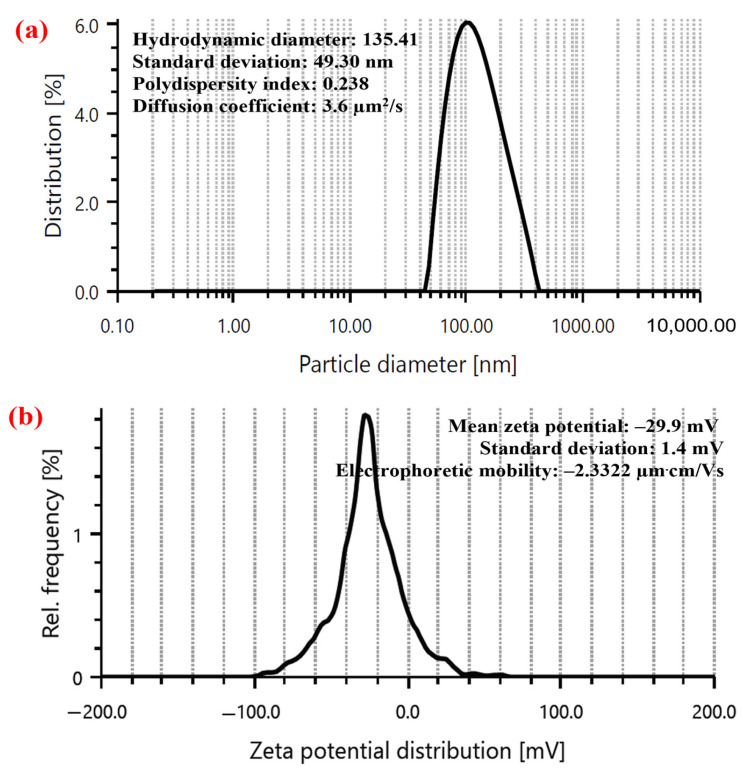
(**a**) DLS and (**b**) ZP of synthesized AgNPs.

Figure 7a presents the PSD and average hydrodynamic diameter of the synthesized AgNPs. The PSD was observed to be in the range of 40 to 400 nm, with the average hydrodynamic size of AgNPs to be at 135.41 ± 49.30 nm. The PDI and diffusion coefficient were observed to be 0.238 and 3.6 µm^2^/s. Al-Otibi et al. [34] reported the PSD to be in the range of 30 to 400 nm with an average particle size of 100.6 nm and a PDI of 0.193. Riyanto et al. [35] reported the PSD to be in the range of 150 to 3000 nm with an average particle size of 332 nm. Chen et al. [21] reported the occurrence of two peaks with PSD in the range of 20 to 50 nm and 60 to 300 nm. The average particle size was at 121 nm with a PDI of 0.386.

#### 3.3.3. Surface Charge

ZP is a key indicator of the stability and behavior of colloidal systems, reflecting the surface charge of NPs in a suspension. It is measured by evaluating the electrophoretic mobility of particles under an applied electric field, which provides insights into interparticle interactions and stability. A higher absolute (>±30 mV) [79] value of zeta potential generally suggests increased stability against aggregation, making it an essential parameter. Figure 7b presents the surface charge of the synthesized AgNPs. The AgNPs showed −29.9 ± 1.4 mV with −2.3322 µm·cm/Vs as electrophoretic mobility. The surface charge of AgNPs determines their electrophoretic mobility, as it influences how the particles move in response to an electric field [80,81,82]. A higher surface charge results in greater electrophoretic mobility, indicating stronger electrostatic interactions and stability in colloidal solutions [83,84].

#### 3.3.4. Crystallinity

**Figure 8:**
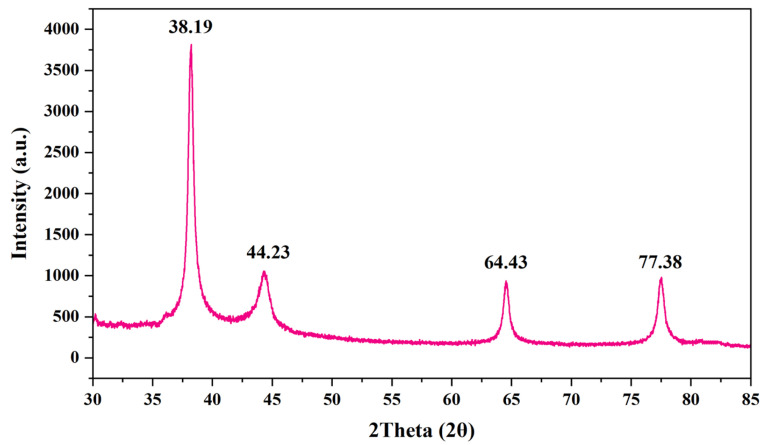
XRD spectra of AgNPs.

XRD is a powerful analytical technique used to determine the crystallographic structure, phase composition, and orientation of materials at the atomic level. By directing X-rays onto a crystalline sample and measuring the resulting diffraction patterns, XRD provides valuable information about the arrangement of atoms, allowing researchers to identify phases and characterize materials. Figure 8 presents the XRD spectra of AgNPs. From the spectra, it was evident that the AgNPs were crystalline in nature with no occurrence of other peaks, indicating the purity of the AgNPs synthesized. A total of four peaks were observed at 38.19°, 44.23°, 64.43°, and 77.38°, whereas the crystallite size was found to be 25.49 nm (using Equation (1)). The observed peaks were indexed to (111), (200), (220), and (311) planes, and the data observed were in correspondence with JCPDS #31-1238 [85].

#### 3.3.5. Morphological Analysis

##### FE-SEM Analysis

**Figure 9:**
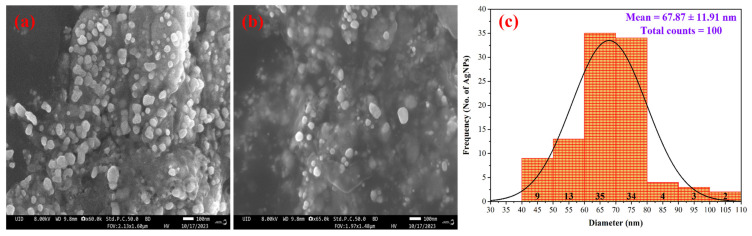
(**a**,**b**) FE-SEM images of synthesized AgNPs at 100 nm scale. (**c**) PSD analysis using histogram (ImageJ Software (ImageJ 1.49v, National Institute of Health, Bethesda, MD, USA)).

AgNPs appeared as well-defined, spherical particles with smooth surfaces when analyzed under FE-SEM (Figure 9a,b). The size of these AgNPs varied in the range of 40 to 110 nm with an average particle size of 67.87 ± 11.91 nm (Figure 9c). The aggregation of AgNPs was observed, which can further influence their potential as antibacterial agents. Furthermore, FE-SEM showed homogeneity of AgNPs, their dispersion on substrates, and showed no potential shape irregularities or defects. AgNPs synthesized from the aqueous extract of *I. cylindrica* showed a spherical shape with a diameter of less than 100 nm [57]. The observed results were in alignment with the reported literature [86,87,88].

##### TEM Analysis

**Figure 10:**
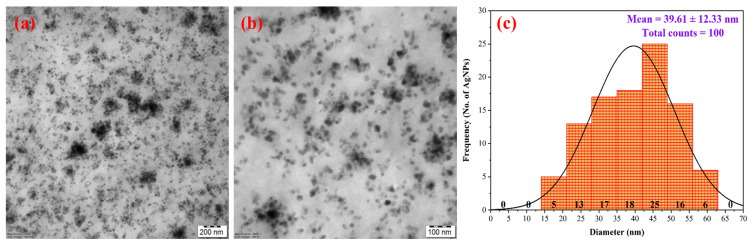
TEM images of synthesized AgNPs at (**a**) 200 nm and (**b**) 100 nm scale. (**c**) PSD analysis using histogram (ImageJ Software (ImageJ 1.49v, National Institute of Health, Bethesda, MD, USA)).

TEM is an essential technique for investigating the morphology and structural properties of NPs. From the analysis, it was evident that the AgNPs were uniform, spherical, and dispersive (Figure 10a,b). Only minimal aggregation was observed. Figure 10c presents the PSD analysis determined using ImageJ software, which was in the range of 15 to 62.5 nm, with an average size of 39.61 ± 12.33 nm. The size determined by the TEM analysis was found to be lower than that of the DLS analysis, which is in alignment with the previous articles [34]. One possible explanation was that DLS measures the hydrodynamic size of AgNPs coated with phytoconstituents on their surface. This causes the scattered light intensity to indicate a larger (and inaccurate) size for the AgNPs. In contrast, TEM determines the true size of the AgNPs [34]. Al-Otibi et al. [34] reported that the synthesized AgNPs were to be spherical and well-dispersed aggregates with sizes in the range of 12 to 59 nm. Chen et al. [21] observed definite spherical-shaped AgNPs with no visible aggregation with a diameter ranging from 17 to 25.8 nm. Keshari et al. [89] reported spherical-shaped and variable -sized AgNPs with sizes ranging from 5 to 35 nm.

#### 3.3.6. Elemental Analysis

**Figure 11:**
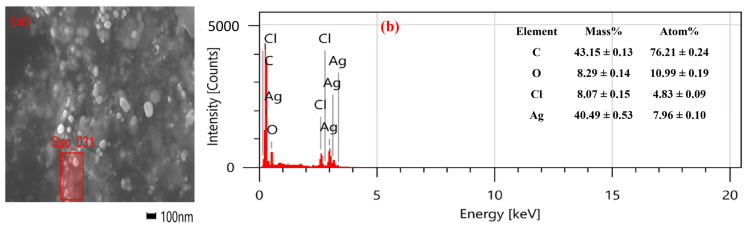
(**a**) FE-SEM image showing EDX sample site and (**b**) EDX spectra.

The EDX spectrum provides qualitative and semi-quantitative insights into the elemental composition, confirming the presence and relative abundance of Ag in the NPs in combination with FE-SEM (Figure 11a). An EDX analysis of AgNPs revealed a strong peak corresponding to the presence of Ag (which was the primary constituent) in the range of 3.0 to 3.5 keV (Figure 11b). However, due to the green synthesis (usage of plant extract) method, additional peaks of oxygen (O), carbon (C), and chlorine (Cl) were observed, which were from plant extract acting as capping agents, stabilizers, or the surrounding environment [90,91]. The observed results were in alignment with the reported literature [91,92,93].

### 3.4. Biomedical Studies

#### 3.4.1. Antibacterial Studies

**Figure 12:**
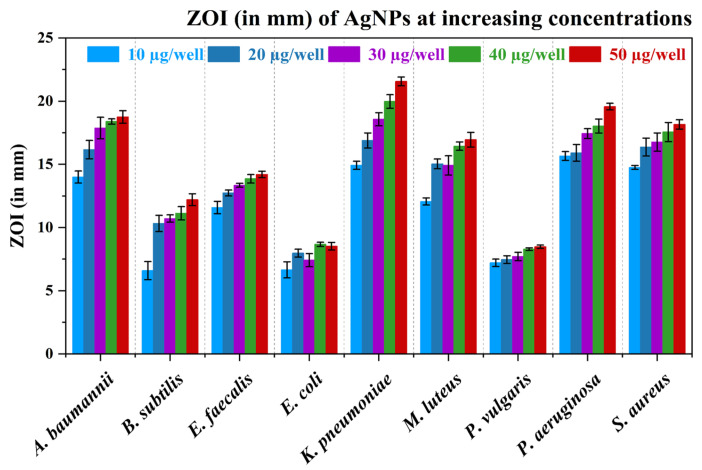
Bar graph showing the ZOI (in mm) of AgNPs at various concentrations against test bacteria. Data are presented as mean (*n* = 3) ± standard deviation.

Figure S2 presents the agar well diffusion assay images of AgNPs at various concentrations against test bacteria. Figure 12 presents a bar graph showing the ZOI (in mm) of AgNPs at various concentrations against test bacteria (Table 4). Gram-negative bacteria (*K. pneumoniae*, *P. aeruginosa*, and *A. baumannii*) showed relatively higher ZOI at lower concentrations, indicating stronger susceptibility to AgNPs compared to other bacteria. Gram-positive bacteria like *S. aureus*, *M. luteus*, and *B. subtilis* also showed significant antibacterial activity, although the ZOI generally increased more gradually with concentration. *E. coli* and *P. vulgaris*, both Gram-negative, exhibited the lowest ZOI values, indicating that they are less affected by the AgNPs compared to other tested bacteria. Overall, both Gram-negative and Gram-positive bacteria showed varying degrees of susceptibility to the AgNPs at various concentrations. The data also suggest that AgNPs are effective antibacterial agents, with varying levels of efficacy depending on the type of bacteria and the concentration used. Gram-negative bacteria tend to be more susceptible to AgNPs, but certain Gram-positive strains also show significant inhibition at higher concentrations.

**Figure 13:**
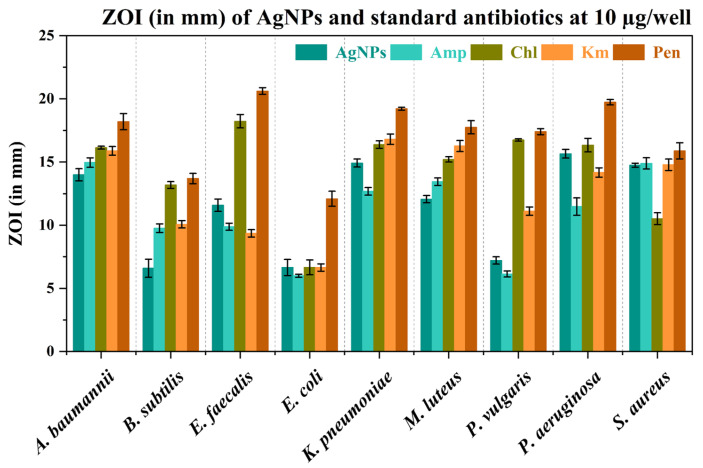
ZOI (in mm) of AgNPs and standard antibiotics at 10 µg/well concentration against test bacteria. Data are presented as mean (*n* = 3) ± standard deviation.

In comparison with standard antibiotics, AgNPs exhibited variable antibacterial activity across different bacterial strains, with some strains responding better than others. In many cases, traditional antibiotics show stronger antibacterial effects compared to AgNPs, particularly with Pen and Chl demonstrating the highest ZOIs across several bacterial species. Figure 13 and Table S1 present the ZOI (in mm) of AgNPs and standard antibiotics (Amp, Chl, Km, and Pen) at 10 µg/well concentration.

The interaction of AgNPs with bacterial membranes is significantly influenced by their surface chemistry and charge. These properties determine how AgNPs interact with the bacterial cell envelope, affecting their antibacterial efficacy. The surface charge, in particular, plays a crucial role in the electrostatic interactions between the AgNPs and the bacterial membranes, which can lead to membrane disruption and bacterial cell death [94]. Positively charged AgNPs exhibit stronger bactericidal activity compared to negatively charged or neutral AgNPs. This is due to the enhanced electrostatic attraction between the positively charged NPs and the negatively charged bacterial cell membranes, facilitating closer contact and more effective membrane disruption [94]. The spatial distribution of cationic functional groups on the NPs’ surface also affects their interaction with bacterial membranes. NPs functionalized with cationic polymers induce significant membrane damage and cyto-toxicity, whereas those with short cationic ligands show minimal impact, highlighting the importance of charge distribution in determining biological interactions [95]. AgNPs can disrupt bacterial membranes by interacting with membrane biomolecules such as lipopolysaccharides and phospholipids. This interaction can lead to structural changes in the membrane, such as breaking phosphodiester bonds and altering the lipid bilayer, resulting in increased membrane permeability and cell lysis [96]. Dendronized AgNPs have been shown to permeabilize bacterial membranes effectively, overcoming barriers like lipopolysaccharides and enhancing the antibacterial effects of other agents, such as lytic enzymes [97]. The primary target of AgNPs in Gram-negative bacteria, such as *E. coli* and *P. aeruginosa*, is the plasma membrane. AgNPs cause depolarization, the leakage of intracellular ions, and the inhibition of cellular respiration, leading to rapid bactericidal effects [98]. AgNPs can also disrupt the bacterial cytoskeleton, as seen with bimetallic NPs, which affect the actin cytoskeleton and lead to morphological changes in bacterial cells [99]. Charge-reversal strategies, where AgNPs switch from negative to positive charge in specific environments, can enhance targeted bacterial killing. This approach allows for the NPs to initially avoid premature interactions and then become highly effective upon reaching the target site [100]. While positively charged AgNPs generally show higher antibacterial activity, the specific surface chemistry and charge distribution are crucial in determining their interactions with bacterial membranes. The effectiveness of AgNPs can be modulated by altering these properties, allowing for targeted and efficient bacterial killing. However, the potential for resistance development and the impact on non-target organisms should be considered when designing AgNP-based antibacterial agents.

#### 3.4.2. Anti-Inflammatory Studies

AgNPs have shown promising potential in anti-inflammatory applications, with one key mechanism being their ability to inhibit protein denaturation, a process linked to inflammation [29]. The egg albumin PrDI assay is a widely used model to assess the anti-inflammatory activity of compounds, as protein denaturation is a common pathway in inflammatory diseases, leading to the formation of autoantigens and promoting tissue damage [101,102]. AgNPs can stabilize proteins and prevent denaturation, thereby reducing inflammation [103], by inhibiting heat- or chemical-induced denaturation of egg albumin, which can be observed as increased turbidity or changes in protein structure [103]. AgNPs, if effective, inhibit this denaturation by stabilizing the protein’s native structure [40]. The extent of inhibition will be measured spectrophotometrically, typically at 660 nm, where reduced absorbance indicates the stronger inhibition of protein denaturation, signifying the anti-inflammatory potential of the AgNPs [41].

The absorbance at 660 nm is used to measure the inhibition of protein denaturation, which is a proxy for anti-inflammatory activity. This wavelength is chosen because it allows for the detection of changes in protein structure that occur during denaturation [104]. The correlation between the absorbance peak at 660 nm and the extent of inhibition of protein denaturation is primarily related to the measurement of anti-inflammatory activity through spectrophotometric analysis [105]. The absorbance at 660 nm is indicative of the structural changes in proteins, which can be inhibited by certain compounds, thereby reducing inflammation. This method is widely used in studies involving plant extracts and other substances to determine their efficacy in preventing protein denaturation, a process linked to inflammation [105].

**Figure 14:**
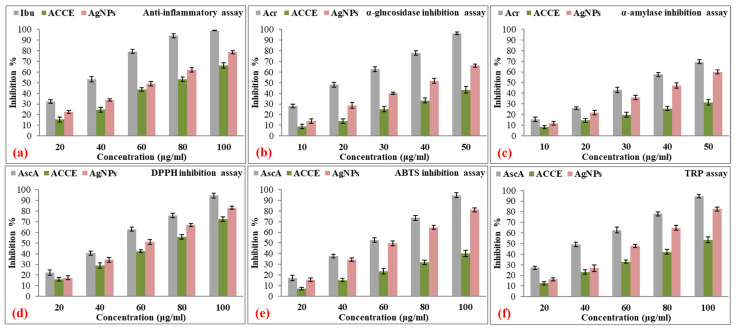
Inhibition percentages (%) of standard (positive control), ACCE (negative control), and AgNPs (sample) at increasing concentrations in (**a**) anti-inflammatory (PrDI), antidiabetic ((**b**) α-glucosidase and (**c**) α-amylase inhibition), and antioxidative ((**d**) DPPH, (**e**) ABTS, and (**f**) TRP) studies. Standards used were Ibuprofen (anti-inflammatory); Acarbose (antidiabetic); and Ascorbic acid (antioxidative).

As the concentration increases, the inhibition for all three samples rises. At 80 µg and 100 µg, the standard approaches complete inhibition, with values of 94.20 ± 1.84% and 99.11 ± 0.24%, respectively. AgNPs also show a significant increase in activity, reaching 62.07 ± 2.08% at 80 µg and 78.62 ± 1.55% at 100 µg. ACCE, while less potent, exhibits a steady rise to 53.27 ± 2.29% at 80 µg and 66.37 ± 2.51% at 100 µg (Figure 14a). The half-maximal inhibitory concentration (IC_50_) values were 37.56 ± 1.65 µg/mL, 75.18 ± 3.15 µg/mL, and 61.08 ± 2.66 µg/mL for standard, ACCE, and AgNPs, respectively (Table 5).

#### 3.4.3. Antidiabetic Studies

##### α-Glucosidase Assay

As the concentration increases, all three samples exhibit a dose-dependent increase in inhibition. At higher concentrations (40 and 50 µg/mL), the standard displayed near-complete inhibition of α-glucosidase, with 78.16 ± 2.08% and 96.33 ± 1.21%, respectively. AgNPs also showed a significant increase, reaching 51.76 ± 2.14% at 40 µg/mL and 66.12 ± 1.56% at 50 µg/mL. ACCE remained the least effective, with inhibition values of 33.36 ± 2.15% and 43.35 ± 3.13% at these respective concentrations (Figure 14b). The IC_50_ values were 20.84 ± 1.02 µg/mL, >100 µg/mL, and 38.69 ± 1.63 µg/mL for standard, ACCE, and AgNPs, respectively (Table 5).

##### α-Amylase Assay

At 50 µg/mL, the standard inhibits α-amylase by 69.61 ± 1.92%, AgNPs by 59.95 ± 2.03%, and ACCE by 31.28 ± 2.68%. AgNPs consistently show stronger inhibition compared to ACCE, indicating they may be a more effective alternative for α-amylase inhibition (Figure 14c). The IC_50_ values were 34.81 ± 1.13 µg/mL, >100 µg/mL, and 42.31 ± 2.24 µg/mL for standard, ACCE, and AgNPs, respectively (Table 5).

#### 3.4.4. Antioxidative Studies

##### DPPH Assay

At higher concentrations of 80 and 100 µg/mL, the standard exhibits near-complete radical scavenging, achieving 75.96 ± 2.23% and 94.68 ± 2.31%, respectively. AgNPs also show strong antioxidant activity at these levels, with 66.93 ± 1.55% inhibition at 80 µg/mL and 83.13 ± 1.41% at 100 µg/mL, outperforming ACCE, which achieves 55.79 ± 2.26% and 72.43 ± 2.03% inhibition at the same concentrations (Figure 14d). The IC_50_ values were 47.60 ± 1.35 µg/mL, 71.78 ± 2.98 µg/mL, and 58.88 ± 2.70 µg/mL for standard, ACCE, and AgNPs, respectively (Table 5).

##### ABTS Assay

At the highest concentration of 100 µg/mL, the standard reaches near-complete inhibition at 94.67 ± 2.38%, and AgNPs also show significant antioxidant activity at 80.97 ± 1.85%. ACCE, while showing some improvement, reaches only 40.12 ± 2.88% inhibition, far lower than both the standard and AgNPs (Figure 14e). The IC_50_ values were 56.90 ± 2.33 µg/mL, >100 µg/mL, and 60.64 ± 2.94 µg/mL for standard, ACCE, and AgNPs, respectively (Table 5).

##### TRP Assay

At the highest concentration of 100 µg/mL, the standard reaches near-complete inhibition at 94.51 ± 1.37%, indicating very strong reducing power. AgNPs also demonstrate significant antioxidant capacity at this concentration, with 82.57 ± 1.84% inhibition, while ACCE reaches only 53.52 ± 2.81%, remaining much lower than the other two (Figure 14f). The IC_50_ values were 40.81 ± 1.80 µg/mL, 93.59 ± 1.97 µg/mL, and 62.84 ± 1.92 µg/mL for standard, ACCE, and AgNPs, respectively (Table 5).

### 3.5. Toxicity Studies

#### 3.5.1. Cyto-Toxicity Assay

The increasing presence of AgNPs has raised concerns about potential cyto-toxic effects on human and animal health [106,107]. The cyto-toxicity of AgNPs has been widely studied in different cell lines, including cancerous and non-cancerous cells, to understand their interaction with biological systems [108,109,110]. Various factors such as particle size, surface charge, concentration, and exposure duration influence AgNPs-induced cyto-toxicity [111,112,113]. Studies have demonstrated that AgNPs can induce oxidative stress, mitochondrial dysfunction, and apoptosis in cell lines, revealing their potential to damage cellular structures [112]. Investigating AgNPs cyto-toxicity across diverse cell lines is crucial for evaluating their safety in biomedical and industrial applications [114,115].

**Figure 15:**
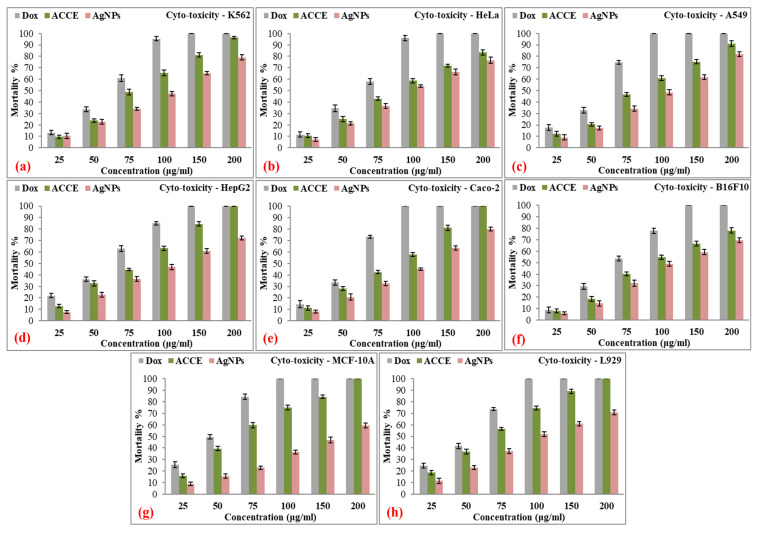
Mortality percentages (%) of different cancerous ((**a**) K562, (**b**) HeLa, (**c**) A549, (**d**) HepG2, (**e**) Caco-2, and (**f**) B16F10) and non-cancerous ((**g**) MCF-10A and (**h**) L929) cell lines at increasing concentrations of Dox (standard; positive control), ACCE (negative control), and AgNPs (sample) after 48 h of exposure.

Overall, Dox consistently shows the highest cyto-toxicity across all cancerous and non-cancerous cell lines, reaching complete mortality at lower concentrations compared to ACCE and AgNPs. ACCE demonstrates moderate toxicity, while AgNPs show the least cyto-toxicity in most cases. However, Dox’s high toxicity toward non-cancerous cells suggests it may cause significant damage to healthy tissues. AgNPs, with lower toxicity in non-cancerous cells, could be a safer alternative, though they are less potent against cancer cells. Figure 15 presents the mortality percentages of various cell lines treated with Dox, ACCE, and AgNPs at varying concentrations (25, 50, 75, 100, 150, and 200 µg/well). The cell lines tested include both cancerous and non-cancerous types, with the objective of comparing the cyto-toxic effects across different concentrations. Table 6 presents the details of the cyto-toxicity studies of Dox, ACCE, and AgNPs against different cell lines at increasing concentrations after 48 h exposure. Table 7 presents the comparison of cyto-toxicity studies of AgNPs against cancerous and non-cancerous cell lines with those reported in the literature.

#### 3.5.2. Eco-Toxicity Assay

The eco-toxicity *Artemia nauplii* assay involving AgNPs is a widely used in vitro method for assessing the environmental risks posed by nanomaterials, particularly in aquatic ecosystems [44]. Due to their widespread use in various industries, AgNPs can be released into water bodies, raising concerns about their potential toxicity to marine organisms [136,137]. *Artemia nauplii*, or brine shrimp larvae, serve as a sensitive bioindicator species in this assay, allowing for the evaluation of AgNPs’ toxicity through mortality and sub-lethal effects [106]. The assay provides insights into how AgNPs may affect aquatic life, contributing to environmental risk assessments and the development of safer nanoparticle technologies [138,139].

**Figure 16:**
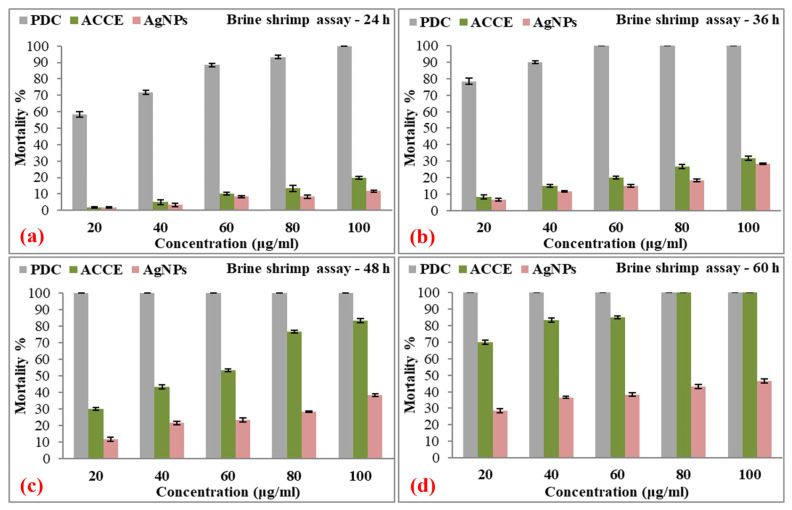
Mortality percentages (%) of *Artemia nauplii* at increasing concentrations of PDC (standard; positive control), ACCE (negative control), and AgNPs (sample) after exposure of (**a**) 24 h, (**b**) 36 h, (**c**) 48 h, and (**d**) 60 h.

Figure 16 presents the mortality percentage of the eco-toxicity *Artemia nauplii* assay, testing different concentrations of AgNPs and controls over a time period of 60 h. For each treatment (positive control (PDC), negative control (ACCE), and AgNPs), the number of dead nauplii and the mortality percentages were reported at intervals of 24 h, 36 h, 48 h, and 60 h.

The results for the AgNPs indicate lower toxicity compared to the controls (both positive and negative) but still show a concentration- and time-dependent increase in *Artemia nauplii* mortality. At the lowest concentration of AgNPs (20 µg/mL), the mortality was 1.67 ± 0.47% at 24 h, gradually increasing to 28.33 ± 1.25% by 60 h. At the highest concentration (100 µg/mL), the mortality rose from 11.67 ± 0.47% at 24 h to 46.67 ± 1.25% at 60 h. Although AgNPs exhibit less toxicity than the positive and negative controls, their increasing mortality rates with higher concentrations and longer exposure times suggest a cumulative toxic effect on *Artemia nauplii*.

The mortality percentages increase with both the concentration of AgNPs and the duration of exposure, suggesting that prolonged exposure or higher concentrations can amplify the harmful effects. The relatively low mortality at early time points (24 to 36 h) compared to controls indicates that AgNPs may exhibit a slower mechanism of action, possibly related to nanoparticle uptake or bioaccumulation within the organism. These findings highlight the importance of considering both concentration and exposure time when assessing the eco-toxicity of AgNPs in aquatic environments. The results also imply that while AgNPs are not immediately lethal at lower concentrations, their chronic exposure could pose a significant ecological risk. Table 8 presents the details of the eco-toxicity assays of PDC, ACCE, and AgNPs at different concentrations and exposure times. Table 9 presents the comparison of AgNPs’ LC_50_ value with those reported in the literature for the brine shrimp eco-toxicity assay.

## 4. Conclusions

Green synthesis employs straightforward procedures, utilizes readily available raw materials, and is environmentally friendly, cost-effective, and scalable for large-scale AgNPs production. Additionally, it does not require high temperatures, pressure, or toxic chemicals. This study focused on the green synthesis of AgNPs using *C. citratus* extract as a reducing agent. Various parameters: the concentration of the Ag precursor and ACCE, the reaction temperature, pH, and time were optimized to control the size and distribution of AgNPs. By determining the influence of various factors, AgNPs can be produced with maximum yield, ensuring efficient resource utilization. The results showed that increasing these parameters generally led to an increase in the yield and size of AgNPs, except for very high concentrations, which caused agglomeration. Characterization techniques like UV-Vis spec, DLS, ZP, XRD, FE-SEM, TEM, and EDX confirmed the successful synthesis and provided detailed insights into the AgNPs’ optical, crystalline, morphological, and elemental properties.

The green synthesis of AgNPs using ACCE represents a promising approach to producing biocompatible AgNPs with enhanced biological properties. In antibacterial studies, the AgNPs demonstrated effective antibacterial activity against both Gram-positive and Gram-negative bacteria, with varying ZOI observed depending on the concentration of AgNPs used. The study also proposed a potential antibacterial mechanism, highlighting how AgNPs interact with bacterial cell membranes, leading to the increased permeability, enzyme inhibition, and disruption of cellular functions. This work underscores the potential of ACCE in the eco-friendly synthesis of AgNPs and their application in antimicrobial treatments. The results demonstrate that the AgNPs exhibit promising inhibitory effects on protein denaturation, α-glucosidase, α-amylase, and antioxidant activity, outperforming ACCE, particularly at higher concentrations. While the standard (positive control) consistently shows superior efficacy across all tests, AgNPs present a viable alternative, especially in their capacity to modulate inflammatory responses and antioxidant activity. The IC_50_ values indicate that AgNPs are more effective than ACCE but less potent than the standard, suggesting their potential as a therapeutic agent. Toxicity (cyto and eco) studies showed that AgNPs were less toxic than standard and ACCE. The present study provides critical insights into the physicochemical characteristics and biological activities of these AgNPs, laying the groundwork for their potential use in a variety of therapeutic applications. As research in this area continues to evolve, it is expected that such green synthesis methods will play a pivotal role in the sustainable development of nanotechnology

## Figures and Tables

**Table 1:** Details of AgNPs synthesized using *C. citratus* extract (Only full-length research articles which are indexed in Scopus were considered. The literature search was conducted on 5 October 2024 via Google Scholar; keywords used for search: “*Cymbopogon citratus*” and “silver nanoparticles”).

Year	Characteristics	Applications	Remarks	Ref.
2024	464 nm, 50.29 nm	Antibacterial	Only one bacterium was used	[31]
2023	431 nm, 43.82 nm, −12.6 mV, 2 to 200 nm, spherical, and well dispersed	Antibacterial and toxicity	Standard was missing in toxicity studies	[32]
2023	410 nm	Antibacterial	Showed minimal activity	[33]
2023	450 nm, 100.6 nm, 12 to 59 nm, spherical, and well dispersed	Antifungal	Similar activity to control	[34]
2022	390 to 470 nm	Antibacterial	Standard was not used	[22]
2022	430 nm, 332 nm	Antifungal	Standard was not used	[35]
2019	~35 nm	Antibacterial and cyto-toxicity	Standard was not used	[36]
2019	435 nm, 121.1 nm, 17 to 25.8 nm, spherical, and well dispersed	Antibacterial, antifungal, and anticarcinogenic	Standard was not used	[21]
2017	435 nm, 10 to 33 nm, spherical, and well dispersed	Antioxidant and antibacterial	Plant extract was not used as control	[20]
2011	430 nm, 32 nm, spherical, and well dispersed	Antibacterial and antifungal	-	[37]

**Table 2:** Wavelength and absorbance values observed during OFAT studies of AgNPs synthesis.

AgNO_3_ Concentration	ACCE Concentration	Temp.	pH	Time	Wavelength	Abs
mM	% *v*/*v*	°C	-	min	nm	a.u.
0.25	3	RT	6.5 to 7.0	30	413.0	0.488
0.50					413.5	0.654
0.75					414.0	0.818
1.00					414.0	0.953
1.25					413.5	1.062
**1.50**					**415.5**	**1.364**
1.75					418.0	1.198
2.00					417.5	1.215
1.50	0.5	RT	6.5 to 7.5	30	413.5	0.454
	1.0				413.5	0.753
	1.5				412.5	0.979
	2.0				414.5	1.130
	2.5				412.5	1.216
	3.0				416.0	1.294
	**3.5**				**414.5**	**1.421**
	4.0				420.5	1.422
1.50	3.5	RT	6.5 to 7.5	30	427.0	0.198
		40			422.0	0.198
		50			422.0	0.258
		60			427.0	0.298
		70			423.0	0.385
		80			417.0	0.440
		90			419.0	0.531
		**100**			**411.0**	**0.633**
1.50	3.5	100	3	30	398.0	0.556
			5		421.0	1.342
			7		417.0	1.410
			**9**		**416.5**	**1.506**
			11		Two peaks	-
1.50	3.5	100	9	0	-	-
				30	423.0	1.489
				**60**	**419.0**	**1.748**
				90	419.5	1.706
				120	421.0	1.638

**Table 3:** A comparison with the reported literature. The literature was chosen based on its relevance to the reported work.

Plant	Parameters	Studied Values	Optimized Value	Ref.
*C. citratus*	AgNO_3_ concentration Extract concentration Reaction temperature Reaction pH Reaction time	0.25, 0.50, 0.75, 1.00, 1.25, 1.50, 1.75, 2.00 mM 0.5, 1.0, 1.5, 2.0, 2.5, 3.0, 3.5, and 4.0% *v*/*v* RT, 40, 50, 60, 70. 80, 90, 100 °C 3, 5, 7, 9, 11 30, 60, 90, 120 min	1.50 mM 3.5% *v*/*v* 100 °C 9 60 min	Present work
*Filipendula ulmaria*	AgNO_3_ concentration Extract concentration Reaction temperature Reaction pH	5, 10, 20 mM 5, 10, 20% *v*/*v* 25, 50, 80 °C 3, 6, 11	10 mM 10% *v*/*v* 25 °C 11	[56]
*Azadirachta indica*	AgNO_3_ concentration Extract concentration Reaction temperature Reaction pH Reaction time	1, 2, 3, 4 mM 1:1, 1:2, 1:3, 1:4, 1:5 ratio RT, 30, 40, 50, 60, 70 °C 5, 7, 9, 11, 13 1, 2, 3, 4, 5 h	2 mM 1:1 ratio 70 °C 11 3 h	[58]
*Ephedra intermedia*	AgNO_3_ concentration Extract concentration Reaction temperature Reaction pH	1.0, 1.5, 2.0, 2.5, 3.0 mM 1:1, 1:2, 1:3, 1:5, 1:7, 1:10 *v*/*v* 30, 45, 60 °C 4, 5, 5.7, 7, 8, 9	2.5 mM 1:10 *v*/*v* 60 °C 8	[59]
*Moringa oleifera*	AgNO_3_ concentration Extract concentration Reaction time	1.0, 1.5, 2.0, 2.5, 3.0 mM 5, 10, 15, 20, 25 mL 20, 60, 240, 720, 1440 min	3 mM 20 mL 1440 min	[60]
Pineapple leaves	AgNO_3_ concentration Extract concentration Reaction time	5, 10, 15, 20, 25 mM 2, 4, 6, 8 mL 2, 4, 6, 24 h	20 mM 6 mL 24 h	[61]
*Laggera tomentosa*	AgNO_3_ concentration Extract concentration Reaction temperature Reaction pH Reaction time	2, 4, 6, 8, 9 mM 15, 20, 30 mL 45, 55, 60, 75, 90 °C 3, 5, 7, 8, 9, 11, 12 20, 30, 40 min	2 mM 20 mL 75 °C 11 20 min	[62]
*Eucalyptus camaldulensis*	AgNO_3_ concentration Extract concentration Reaction temperature Reaction pH Reaction time	0.5, 1.0, 1.5, 2.0 mM 1:9, 2:8, 3:7, 4:6, 5:5 *v*/*v* 25, 50, 75, 100 °C 1, 3, 5, 7, 9, 11 15, 30, 45, 60 min	1 mM 3:7 *v*/*v* 75 °C 7 60 min	[57]
*Imperata cylindrica*	AgNO_3_ concentration Extract concentration Reaction temperature Reaction pH	5, 10 mM 10, 50% 30, 60, 100 °C 3, 7, 10	10 mM 10% 60 °C 5.7	[63]
*Citrus limon*	AgNO_3_ concentration Extract concentration Reaction time	0.5, 1, 2 mM 1:9, 2:8, 5:5 *v*/*v* 30, 60, 120, 180, 240 min	1 mM 1:9 *v*/*v* 240 min	[64]
*Teucrium polium*	AgNO_3_ concentration Extract concentration Reaction temperature Reaction pH Reaction time	1, 5, 10, 15, 20, 25, 50 mM 100, 150, 300, 600, 900 µL 30, 60, 90 °C 1, 2, 3, 4, 5, 6, 7, 8, 9, 10, 11, 12, 13 5, 10, 15, 20, 25, 30 min	15 mM 300 µL 30 °C 6 15 min	[65]

**Table 4:** ZOI (in mm) of AgNPs at various concentrations against the test bacteria. Data are presented as mean (*n* = 3) ± standard deviation. ^a^ indicates *p* > 0.05, ^b^ indicates *p* > 0.005, and ^c^ indicates *p* > 0.0005.

Bacteria	Gram −/+	Concentration (µg/Well)				
		10	20	30	40	50
*A. baumannii*	Gram-negative	13.99 ± 0.48	16.17 ± 0.72 ^c^	17.87 ± 0.85 ^c^	18.39 ± 0.21 ^a^	18.75 ± 0.49 ^a^
*B. subtilis*	Gram-positive	6.59 ± 0.71	10.33 ± 0.64 ^c^	10.71 ± 0.29 ^a^	11.13 ± 0.53 ^a^	12.20 ± 0.47 ^b^
*E. faecalis*	Gram-positive	11.58 ± 0.49	12.74 ± 0.23 ^b^	13.34 ± 0.16 ^a^	13.86 ± 0.33 ^a^	14.20 ± 0.26
*E. coli*	Gram-negative	6.65 ± 0.64	7.97 ± 0.31 ^b^	7.42 ± 0.52	8.67 ± 0.17 ^b^	8.52 ± 0.30
*K. pneumoniae*	Gram-negative	14.92 ± 0.32	16.89 ± 0.59 ^c^	18.56 ± 0.52 ^c^	19.98 ± 0.53 ^c^	21.56 ± 0.35 ^c^
*M. luteus*	Gram-positive	12.06 ± 0.28	15.02 ± 0.39 ^c^	14.91 ± 0.76	16.44 ± 0.33 ^c^	16.95 ± 0.59 ^a^
*P. vulgaris*	Gram-negative	7.21 ± 0.29	7.47 ± 0.30	7.71 ± 0.33	8.29 ± 0.11 ^a^	8.48 ± 0.13
*P. aeruginosa*	Gram-negative	15.66 ± 0.35	15.91 ± 0.66	17.43 ± 0.39 ^b^	18.03 ± 0.55 ^a^	19.57 ± 0.26 ^c^
*S. aureus*	Gram-positive	14.75 ± 0.16	16.37 ± 0.71 ^c^	16.76 ± 0.73 ^a^	17.56 ± 0.76 ^b^	18.16 ± 0.37 ^a^

**Table 5:** Details of anti-inflammatory (protein denaturation inhibition), antidiabetic (α-glucosidase and α-amylase inhibition), and antioxidative (DPPH, ABTS, and TRP) studies of standard (positive control), ACCE (negative control), and AgNPs at increasing concentrations. Standards used were Ibuprofen (anti-inflammatory); Acarbose (antidiabetic); and Ascorbic acid (antioxidative). Data are presented as mean (*n* = 3) ± standard deviation. ^a^ indicates *p* > 0.05, ^b^ indicates *p* > 0.005, and ^c^ indicates *p* > 0.0005. AgNPs was compared with ACCE.

Assay	Concentration	Inhibition Percentage (%)		
	µg/mL	Standard (Positive Control)	ACCE (Negative Control)	AgNPs (Sample)
Anti-inflammatory (PrDI)	20	32.34 ± 1.76	15.37 ± 2.22	22.60 ± 1.47 ^a^
	40	53.32 ± 2.37	24.57 ± 2.11	33.75 ± 1.12 ^a^
	60	79.42 ± 1.92	43.58 ± 1.53	49.17 ± 2.13 ^a^
	80	94.20 ± 1.84	53.27 ± 2.29	62.07 ± 2.08 ^b^
	100	99.11 ± 0.24	66.37 ± 2.51	78.62 ± 1.55 ^b^
	IC_50_	37.56 ± 1.65	75.18 ± 3.15	61.08 ± 2.66 ^b^
Antidiabetic (α-glucosidase inhibition)	10	28.03 ± 1.75	8.82 ± 1.94	13.80 ± 2.13 ^a^
	20	48.05 ± 2.33	13.94 ± 2.15	28.64 ± 2.71 ^b^
	30	62.70 ± 2.41	25.07 ± 2.44	40.14 ± 1.12 ^b^
	40	78.16 ± 2.08	33.36 ± 2.15	51.76 ± 2.14 ^b^
	50	96.33 ± 1.21	43.35 ± 3.13	66.12 ± 1.56 ^c^
	IC_50_	20.84 ± 1.02	>100	38.69 ± 1.63 ^c^
Antidiabetic (α-amylase inhibition)	10	15.42 ± 2.16	8.04 ± 1.47	11.76 ± 1.72 ^a^
	20	25.87 ± 1.43	14.36 ± 1.99	21.62 ± 2.11 ^b^
	30	42.98 ± 2.46	19.69 ± 2.35	35.89 ± 1.98 ^b^
	40	57.50 ± 1.85	25.47 ± 1.87	47.35 ± 2.47 ^c^
	50	69.61 ± 1.92	31.28 ± 2.68	59.95 ± 2.03 ^c^
	IC_50_	34.81 ± 1.13	>100	42.31 ± 2.24 ^c^
Antioxidative (DPPH)	20	22.13 ± 2.45	15.80 ± 1.72	17.42 ± 2.04
	40	40.60 ± 1.93	28.97 ± 2.43	34.26 ± 2.36 ^a^
	60	63.06 ± 1.82	42.37 ± 1.19	51.02 ± 2.29 ^a^
	80	75.96 ± 2.23	55.79 ± 2.26	66.93 ± 1.55 ^b^
	100	94.68 ± 2.31	72.43 ± 2.03	83.13 ± 1.41 ^b^
	IC_50_	47.60 ± 1.35	71.78 ± 2.98	58.88 ± 2.70 ^c^
Antioxidative (ABTS)	20	16.94 ± 2.47	7.02 ± 1.19	15.44 ± 1.53 ^a^
	40	37.53 ± 1.61	15.09 ± 1.36	34.14 ± 1.74 ^b^
	60	52.78 ± 2.12	23.23 ± 2.51	49.55 ± 2.38 ^c^
	80	73.52 ± 2.27	31.62 ± 2.13	64.59 ± 2.04 ^c^
	100	94.67 ± 2.38	40.12 ± 2.88	80.97 ± 1.85 ^c^
	IC_50_	56.90 ± 2.33	>100	60.64 ± 2.94 ^c^
Antioxidative (TRP)	20	27.15 ± 1.46	12.55 ± 1.47	16.25 ± 1.39
	40	49.07 ± 2.20	23.04 ± 2.16	26.87 ± 3.13
	60	62.72 ± 2.58	32.97 ± 1.59	47.77 ± 1.46 ^b^
	80	77.86 ± 2.03	42.23 ± 2.37	64.75 ± 2.27 ^c^
	100	94.51 ± 1.37	53.52 ± 2.81	82.57 ± 1.84 ^c^
	IC_50_	40.81 ± 1.80	93.59 ± 1.97	62.84 ± 1.92 ^c^

**Table 6:** Details of cyto-toxicity studies of Dox (standard; positive control), ACCE (negative control), and AgNPs (sample) against different cell lines at increasing concentrations after 48 h exposure. Data are presented as mean (*n* = 3) ± standard deviation. ^a^ indicates *p* > 0.05, ^b^ indicates *p* > 0.005, and ^c^ indicates *p* > 0.0005. AgNPs was compared with Dox.

Cell line	Concentration	Mortality percentage (%)		
Type	µg/mL	Standard (Positive Control)	ACCE (Negative Control)	AgNPs (Sample)
K562 (human myelogenous leukemia cell line)	25	13.17 ± 1.93	9.71 ± 1.49	10.52 ± 2.23 ^a^
	50	33.60 ± 2.01	23.89 ± 1.48	22.76 ± 1.96 ^b^
	75	60.76 ± 2.92	48.86 ± 2.46	33.99 ± 1.18 ^c^
	100	95.34 ± 1.84	65.45 ± 2.54	47.36 ± 1.95 ^c^
	150	100.00 ± 0.00	81.16 ± 1.87	65.15 ± 1.48 ^c^
	200	100.00 ± 0.00	96.36 ± 1.14	79.02 ± 2.26 ^c^
	IC_50_	61.86 ± 2.24	76.95 ± 2.58	105.75 ± 1.28 ^c^
HeLa (human cervical cell line)	25	11.57 ± 2.37	10.66 ± 1.67	7.14 ± 1.66 ^a^
	50	34.38 ± 2.91	25.20 ± 2.32	21.38 ± 1.52 ^b^
	75	58.00 ± 2.67	43.05 ± 1.52	36.59 ± 2.11 ^b^
	100	96.08 ± 2.33	58.61 ± 1.96	53.94 ± 1.18 ^c^
	150	100.00 ± 0.00	71.94 ± 1.24	66.36 ± 2.34 ^c^
	200	100.00 ± 0.00	83.34 ± 2.21	76.59 ± 2.58 ^c^
	IC_50_	64.80 ± 2.37	85.40 ± 1.44	92.75 ± 1.26 ^c^
A549 (human adenocarcinomic alveolar basal epithelial cell line)	25	17.81 ± 2.46	12.23 ± 2.23	9.08 ± 2.26 ^b^
	50	32.71 ± 2.51	20.44 ± 1.28	17.21 ± 1.63 ^c^
	75	74.73 ± 1.59	46.65 ± 1.76	34.15 ± 2.34 ^c^
	100	100.00 ± 0.00	60.94 ± 2.17	48.33 ± 2.29 ^c^
	150	100.00 ± 0.00	75.21 ± 1.95	61.71 ± 1.96 ^c^
	200	100.00 ± 0.00	91.07 ± 2.59	81.99 ± 2.12 ^c^
	IC_50_	50.20 ±1.31	82.15 ± 2.06	103.68 ± 1.92 ^c^
HepG2 (human hepatocellular carcinoma cell line)	25	21.94 ± 1.86	12.83 ± 1.42	7.71 ± 1.29 ^b^
	50	36.27 ± 2.03	32.56 ± 2.12	22.75 ± 2.05 ^b^
	75	62.81 ± 2.55	44.65 ± 1.15	36.63 ± 1.99 ^c^
	100	85.09 ± 1.48	63.15 ± 2.01	46.83 ± 2.35 ^c^
	150	100.00 ± 0.00	84.50 ± 1.92	60.78 ± 2.13 ^c^
	200	100.00 ± 0.00	100.00 ± 0.00	72.32 ± 1.66 ^c^
	IC_50_	59.81 ± 1.96	79.26 ± 2.12	107.04 ± 2.67 ^c^
Caco-2 (human colorectal adenocarcinoma cell line)	25	14.45 ± 3.12	11.23 ± 1.89	8.13 ± 1.27 ^a^
	50	33.38 ± 2.38	28.11 ± 1.52	20.65 ± 2.72 ^a^
	75	73.50 ± 1.25	42.58 ± 1.41	32.56 ± 1.89 ^c^
	100	100.00 ± 0.00	57.87 ± 1.74	45.16 ± 1.13 ^c^
	150	100.00 ± 0.00	81.20 ± 2.39	63.56 ± 1.85 ^c^
	200	100.00 ± 0.00	100.00 ± 0.00	80.17 ± 1.64 ^c^
	IC_50_	51.03 ± 1.08	86.48 ± 2.26	110.78 ± 1.45 ^c^
B16 F10 (murine melanoma cell line)	25	8.87 ± 2.57	8.05 ± 1.55	5.98 ± 1.28
	50	15.92 ± 1.59	18.44 ± 2.18	14.49 ± 2.34
	75	53.75 ± 2.04	40.42 ± 1.67	32.13 ± 2.63 ^c^
	100	77.75 ± 2.21	54.79 ± 1.99	48.94 ± 2.06 ^c^
	150	100.00 ± 0.00	66.61 ± 2.03	59.30 ± 2.39 ^c^
	200	100.00 ± 0.00	78.10 ± 2.27	69.46 ± 2.17 ^c^
	IC_50_	69.87 ± 1.65	91.38 ± 2.11	102.35 ± 2.27 ^c^
MCF-10A (non-canceours breast epithelial cell line)	25	25.47 ± 2.47	15.73 ± 1.68	8.97 ± 1.61 ^a^
	50	49.62 ± 2.06	39.49 ± 1.92	15.66 ± 1.99 ^c^
	75	84.32 ± 2.37	59.75 ± 2.31	22.90 ± 1.53 ^c^
	100	100.00 ± 0.00	75.14 ± 2.06	36.53 ± 1.76 ^c^
	150	100.00 ± 0.00	84.39 ± 1.63	46.82 ± 2.51 ^c^
	200	100.00 ± 0.00	100.00 ± 0.00	59.48 ± 2.28 ^c^
	IC_50_	50.47 ± 1.92	62.86 ± 2.43	160.67 ± 2.79 ^c^
L929 (normal murine cell line)	25	24.68 ± 1.94	18.64 ± 1.81	11.56 ± 2.23 ^b^
	50	41.67 ± 2.25	36.67 ± 2.15	22.96 ± 1.56 ^c^
	75	73.79 ± 1.18	56.48 ± 1.38	37.10 ± 2.17 ^c^
	100	100.00 ± 0.00	74.44 ± 1.56	51.85 ± 2.24 ^c^
	150	100.00 ± 0.00	89.15 ± 1.92	61.06 ± 1.84 ^c^
	200	100.00 ± 0.00	100.00 ± 0.00	70.87 ± 2.07 ^c^
	IC_50_	50.83 ± 1.41	66.43 ± 1.95	96.60 ± 1.98 ^c^

**Table 7:** Comparison of cyto-toxicity studies of AgNPs against cancerous and non-cancerous cell lines with those reported in the literature.

Cell Line	Cell Number/Well	Duration	Concentration Range	IC_50_ Value	Ref.
K562	~20,000	48 h	25 to 200 µg/mL	105.75	Present work
HeLa	~20,000	48 h	25 to 200 µg/mL	92.75	
A549	~20,000	48 h	25 to 200 µg/mL	103.68	
HepG2	~20,000	48 h	25 to 200 µg/mL	107.04	
Caco-2	~20,000	48 h	25 to 200 µg/mL	110.78	
B16F10	~20,000	48 h	25 to 200 µg/mL	102.35	
MCF-10A	~20,000	48 h	25 to 200 µg/mL	160.67	
L929	~20,000	48 h	25 to 200 µg/mL	96.60	
K562	~10,000	24 h	1 to 500 µg/mL	50	[116]
K562	~20,000	24 h	6.25 to 50 µg/mL	19.50	[117]
HeLa	~100,000	24 h	6.5 to 100 µg/mL	47.58	[118]
HeLa	~10,000	24 h	10 to 100 µg/mL	56	[90]
HeLa	~10,000	24 h	0.005 to 2.5 µL/mL	1.98	[119]
HeLa	~5000	24 h	0.5 to 10 µg/mL	4.97	[120]
A549	~100,000	24 h	20 to 100 µg/mL	76.90	[121]
A549	~10,000	24 h	1 to 100 µg/mL	9.27	[122]
A549	~20,000	24 h	5 to 50 µg/mL	~50	[123]
HepG2	~7000	24 h	31.25 to 1000 µg/mL	32	[124]
HepG2	~20,000	24 h	2 to 200 µg/mL	5.18	[125]
HepG2	~5000	24 h	10 to 50 µg/mL	37.98	[126]
HepG2	~5000	24 h	0.5 to 10 µg/mL	3.89	[120]
Caco-2	~7000	24 h	31.25 to 1000 µg/mL	90	[124]
Caco-2	~20,000	24 h	12.5 to 200 µg/mL	49.14	[127]
Caco-2	~10,000	24 h	10 to 100 µg/mL	41.59	[128]
B16F10	~20,000	48 h	6.25 to 100 µg/mL	20.2	[129]
B16F10	~5000	24 h	0.5 to 10 µg/mL	3.29	[120]
B16F10	~5000	24 h	10 to 100 µg/mL	36.63	[130]
MCF-10A	~10,000	48 h	0.1 to 50 µg/mL	Not affected	[131]
MCF-10A	~5000	48 h	0.18 to 360 µg/mL	57.97	[132]
MCF-10A	~5000	48 h	0.14 to 288 µg/mL	40.63	[132]
L929	~10,000	24 h	0.0156 to 1 mg/mL	>1	[133]
L929	~100,000	24 h	20 to 1000 µg/mL	~750	[134]
L929	~10,000	24 h	0.0625 to 1 mg/mL	>1	[135]

**Table 8:** Details of eco-toxicity assay of PDC (standard; positive control), ACCE (negative control), and AgNPs at different concentrations and exposure times (initial number of *Artemia nauplii* was 60). Data are presented as mean (*n* = 3) ± standard deviation. ^c^ indicates *p* > 0.0005. AgNPs was compared with PDC.

Concentration (µg/mL)	Number of Dead at (Mean)				Mortality Percentage (%; Mean ± SD) at			
	24 h	36 h	48 h	60 h	24 h	36 h	48 h	60 h
**PDC (Positive Control)**								
Blank	0	2	4	10	0.00 ± 0.00	0.0 ± 0.00	0.00 ± 0.00	0.00 ± 0.00
20	35	47	60	60	58.33 ± 1.70	78.33 ± 1.89	100.00 ± 0.00	100.00 ± 0.00
40	43	54	60	60	71.67 ± 1.25	90.00 ± 0.82	100.00 ± 0.00	100.00 ± 0.00
60	53	60	60	60	88.33 ± 1.25	100.00 ± 0.00	100.00 ± 0.00	100.00 ± 0.00
80	56	60	60	60	93.33 ± 0.94	100.00 ± 0.00	100.00 ± 0.00	100.00 ± 0.00
100	60	60	60	60	100.00 ± 0.00	100.00 ± 0.00	100.00 ± 0.00	100.00 ± 0.00
**ACCE (Negative Control)**								
20	1	5	18	42	1.67 ± 0.47	8.33 ± 1.25	30.00 ± 0.82	70.00 ± 1.41
40	3	9	26	50	5.00 ± 1.41	15.00 ± 0.82	43.33 ± 1.25	83.33 ± 1.25
60	6	12	32	51	10.00 ± 0.82	20.00 ± 0.82	53.33 ± 0.94	85.00 ± 0.82
80	8	16	46	60	13.33 ± 1.70	26.67 ± 1.25	76.76 ± 0.94	100.00 ± 0.00
100	12	19	50	60	20.00 ± 0.82	31.67 ± 1.25	83.33 ± 1.25	100.00 ± 0.00
**AgNPs (Sample)**								
20	1	4	7	17	1.67 ± 0.47 ^c^	6.67 ± 0.94 ^c^	11.67 ± 1.25 ^c^	28.33 ± 1.25 ^c^
40	2	7	13	22	3.33 ± 0.94 ^c^	11.67 ± 0.47 ^c^	21.67 ± 0.94 ^c^	36.67 ± 0.47 ^c^
60	5	9	14	23	8.33 ± 0.47 ^c^	15.00 ± 0.82 ^c^	23.33 ± 1.25 ^c^	38.33 ± 0.94 ^c^
80	5	11	17	26	8.33 ± 0.94 ^c^	18.33 ± 0.94 ^c^	28.33 ± 0.47 ^c^	43.33 ± 1.25 ^c^
100	7	17	23	28	11.67 ± 0.47 ^c^	28.33 ± 0.47 ^c^	38.33 ± 0.94 ^c^	46.67 ± 1.25 ^c^

**Table 9:** Comparison of AgNPs’ LC_50_ value with those reported in the literature for brine shrimp eco-toxicity assay.

Source	Concentration Range	LC_50_ Value	Time	Ref.
*C. citratus*	20 to 100 µg/mL	>100	60 h	Present work
*Pandanus tectorius*	3 to 15 µg/mL	~11	24 h	[118]
*Elaeocarpus serratus*	3 to 15 µg/mL	~12	24 h	[140]
*Pandanus canaranus*	2 to 10 µg/mL	~9	24 h	[121]
*Caulerpa sertularioides*	25 to 200 µg/mL	~150	48 h	[140]
*Senna alexandrina*	5 to 30 µg/mL	<25	24 h	[141]
*Osbeckia leschenaultiana*	20 to 100 µg/mL	175.80	24 h	[142]
*Cladophora fascicularis*	25 to 150 µg/mL	>150	72 h	[143]
*Spondias pinnata*	20 to 320 µg/mL	~30	24 h	[144]
*Padina gymnospora*	10 to 150 µg/mL	47.92	48 h	[145]
*Ipomoea carnea*	100 to 2000 ng/mL	1359.03	24 h	[146]

## Data Availability

The data presented in this study are available on request from the corresponding author.

## Supplementary Materials

The following supporting information can be downloaded at: https://www.mdpi.com/article/10.3390/nano15050328/s1, Figure S1: Pictures representing AgNPs synthesis via OFAT study; Figure S2. Agar well diffusion assay images of AgNPs at various concentrations against test bacteria; Table S1: ZOI (in mm) of AgNPs and standard antibiotics at 10 µg/well concentration against test bacteria.

## List of Abbreviations

ABTS: 2,2′-azino-bis(3-ethylbenzothiazoline-6-sulfonic acid; ACCE: Aqueous *Cymbopogon citratus* extract; Ag: Silver; AgNO_3_: Silver nitrate; AgNPs: Silver nanoparticles; Amp: Ampicillin; AscA: Ascorbic acid; Chl: Chloramphenicol; DLS: Dynamic light scattering; DNS: Dinitrosalicylic acid; DPPH: 2,2-diphenyl-1-picrylhydrazyl; Dox: Doxorubicin; DW: Sterile double-distilled water; EDX: Energy-dispersive X-ray spectroscopy; FE-SEM: Field emission scanning electron microscope; FWHM: Full width at half maximum; HCl: Hydrochloric acid; Ibu: Ibuprofen; Km: Kanamycin; MTT: 3-(4,5-dimethylthiazol-2-yl)-2,5-diphenyltetrazolium bromide; NaOH: Sodium hydroxide; NPs: Nanoparticles; OFAT: One-factor-at-a-time; PrDI: Protein denaturation inhibition; pNPG: p-nitrophenyl-α-D-glucopyranoside; PDI: Polydispersity index; Pen: Penicillin; PSD: Particle size distribution; ROS: Reactive oxygen species; RT: Room temperature; TEM: Transmission electron microscopy; TRP: Total reducing power; UV-Vis spec: Ultraviolet–visible spectroscopy; XRD: X-ray diffraction; ZOI: Zone of inhibition; ZP: Zeta potential.
