# Supplementary material for: Aqueous Cymbopogon citratus Extract Mediated Silver Nanoparticles: Part I. Influence of Synthesis Parameters, Characterization, and Biomedical Studies
Source: Nanomaterials (Basel). 2025 Feb 20;15(5):328. doi: 10.3390/nano15050328 (PMC11901631; doi:10.3390/nano15050328)
Supplement: Supplementary file 1 [file nanomaterials-15-00328-s001.zip › nanomaterials-3458848-supplementary.pdf]

Supplementary Materials

Aqueous *Cymbopogon citratus* Extract Mediated Silver Nanoparticles: Part I. Influence of Synthesis Parameters, Characterization, and Biomedical Studies

Himabindu Kurra [1,2], Aditya Velidandi [3], Mounika Sarvepalli [3], Ninian Prem Prashanth Pabbathi [3] and Vikram Godishala [1,2,*]

[1] Department of Biotechnology, Vaagdevi Degree and P.G. College, Warangal, Telangana 506001, India;
bindukurra21983@gmail.com
[2] Department of Biotechnology, Bharatiya Engineering Science and Technology Innovation University, Gorantla, Andhra Pradesh 515231, India
[3] Department of Biotechnology, National Institute of Technology, Warangal, Telangana 506004, India;
aditya.velidandi@gmail.com (A.V.); mouni.sarvepalli@gmail.com (M.S.); ninian86@gmail.com (N.P.P.P.)
* Correspondence: vikramgodishala@gmail.com

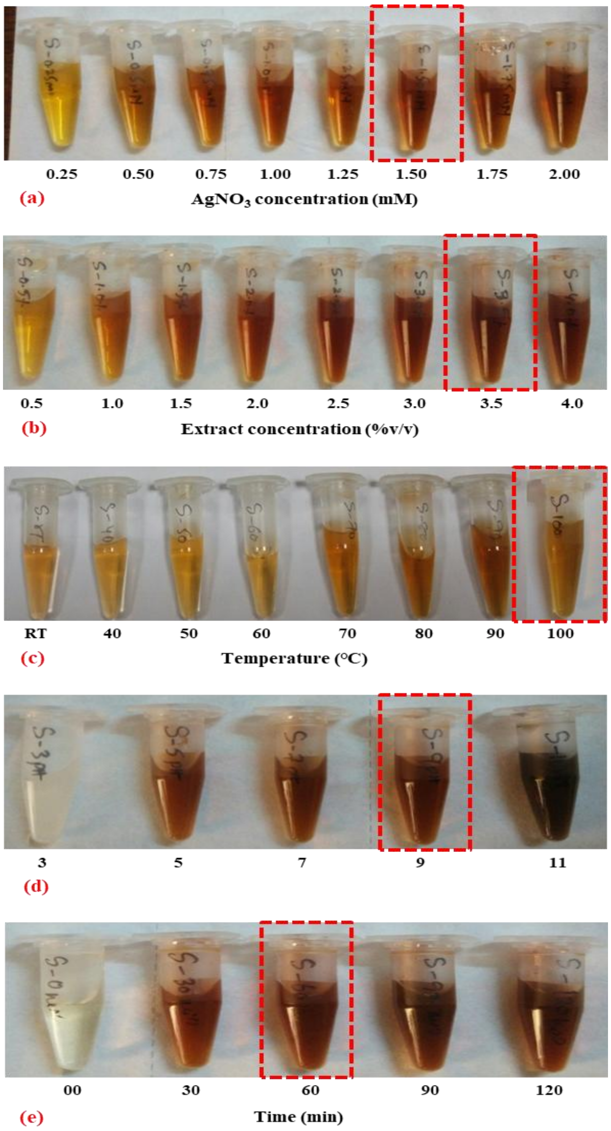

Figure S1. Pictures representing AgNPs synthesis via OFAT study. **(a)** Picture representing AgNPs synthesis at various metal precursor ($AgNO_3$) concentrations. Reaction conditions: $AgNO_3$ concentration—0.25, 0.50, 0.75, 1.00, 1.25, 1.50, 1.75, and 2.00 mM; ACCE concentration—3.0% *v/v*; Reaction temperature—RT; Reaction pH—6.5 to 7.0; and Reaction time—30 min. **(b)** Picture representing AgNPs synthesis at various extract (ACCE) concentrations. Reaction conditions: $AgNO_3$ concentration—1.50 mM; ACCE concentration—0.5, 1.0, 1.5, 2.0, 2.5, 3.0, 3.5, and 4.0% *v/v*; Reaction temperature—RT; Reaction pH—6.5 to 7.0; and Reaction time—30 min. **(c)** Picture representing AgNPs synthesis at various reaction temperatures. Reaction conditions: $AgNO_3$ concentration—1.50 mM; ACCE concentration - 3.5% v/v; Reaction temperature—RT, 40, 50, 60, 70, 80, 90, and 100 °C; Reaction pH—6.5 to 7.0; and Reaction time—30 min. **(d)** Picture representing AgNPs synthesis at various reaction pH. Reaction conditions: $AgNO_3$ concentration—1.50 mM; ACCE concentration—3.5% *v/v*; Reaction temperature - 100 °C; Reaction pH—3, 5, 7, 9, and 11; and Reaction time—30 min. **(e)** Picture representing AgNPs synthesis at various reaction times. Reaction conditions: $AgNO_3$ concentration—1.50 mM; ACCE concentration—3.5% *v/v*; Reaction temperature—100 °C; Reaction pH—9; and Reaction time—0, 30, 60, 90, and 120 min.

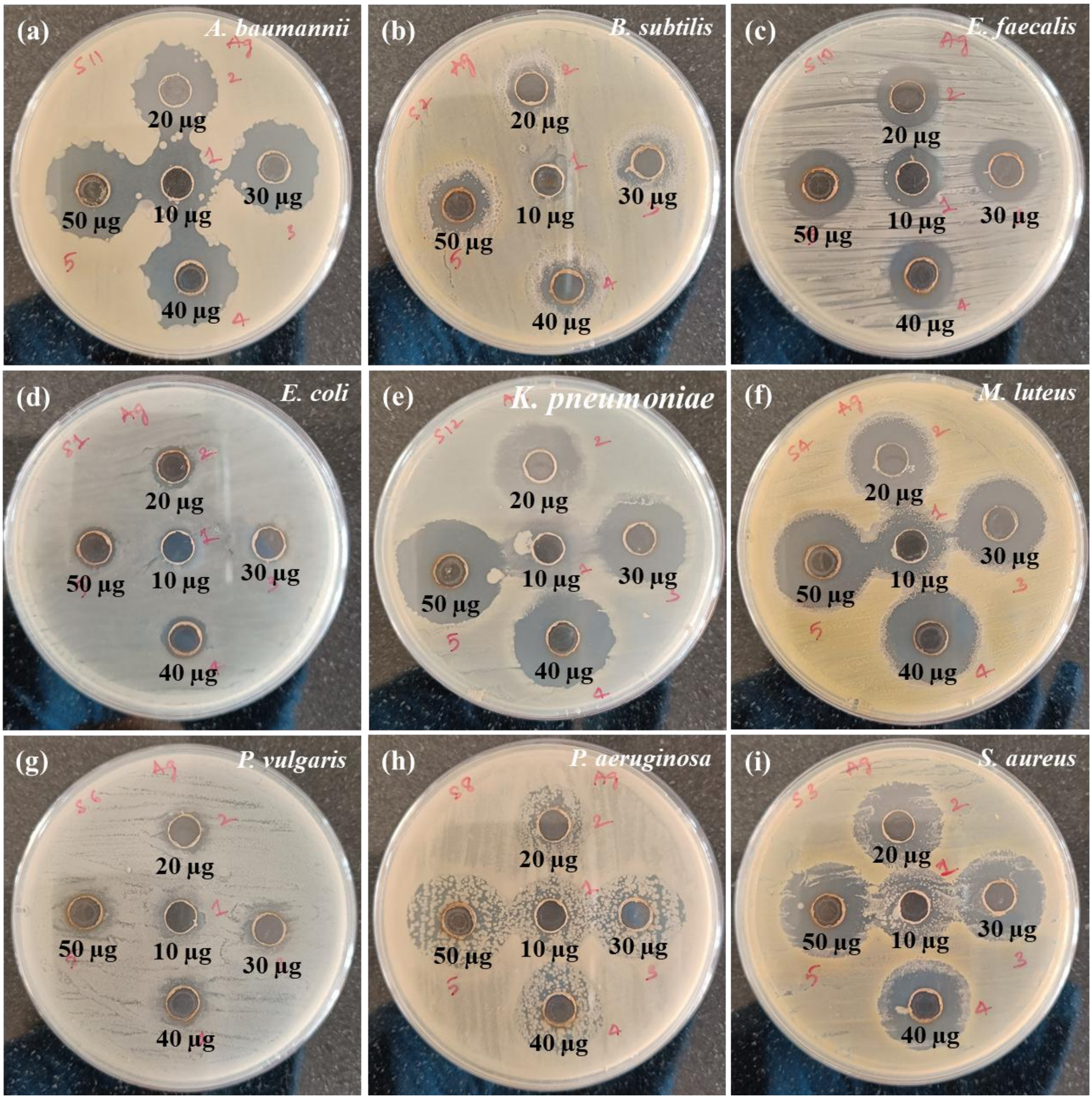

Figure S2. Agar well diffusion assay images of AgNPs at various concentrations against test bacteria.

Table S1. ZOI (in mm) of AgNPs and standard antibiotics at 10 μg/well concentration against test bacteria. Data is presented as mean (n = 3) ± standard deviation.

**Bacteria**	**ZOI (in mm) at 10 μg/well**				
	**AgNPs**	**Amp**	**Chl**	**Km**	**Pen**
A. baumannii	13.99 ± 0.48	14.96 ± 0.37	16.14 ± 0.11	15.89 ± 0.34	18.20 ± 0.64
B. subtilis	6.59 ± 0.71	9.77 ± 0.34	13.18 ± 0.27	10.07 ± 0.29	13.70 ± 0.41
E. faecalis	11.58 ± 0.49	9.88 ± 0.28	18.23 ± 0.53	9.36 ± 0.29	20.62 ± 0.27
E. coli	6.65 ± 0.64	6.00 ± 0.12	6.66 ± 0.59	6.64 ± 0.29	12.09 ± 0.59
K. pneumoniae	14.92 ± 0.32	12.68 ± 0.30	16.38 ± 0.30	16.81 ± 0.41	19.22 ± 0.12
M. luteus	12.06 ± 0.28	13.45 ± 0.30	15.21 ± 0.21	16.27 ± 0.44	17.75 ± 0.52
P. vulgaris	7.21 ± 0.29	6.14 ± 0.24	16.75 ± 0.10	11.12 ± 0.33	17.41 ± 0.24
P. aeruginosa	15.66 ± 0.35	11.47 ± 0.69	16.34 ± 0.53	14.17 ± 0.37	19.74 ± 0.21
S. aureus	14.75 ± 0.16	14.89 ± 0.45	10.52 ± 0.47	14.78 ± 0.45	15.88 ± 0.64
